# A review of *Rhododendron molle*: traditional uses, clinical applications, phytochemistry, pharmacology, toxicology, pharmacokinetics and quality control

**DOI:** 10.3389/fphar.2026.1772696

**Published:** 2026-05-12

**Authors:** Xiaohong Guo, Lijuan Wang, Qiang Ran, Yanyan Li, Jing Leng, Ping Tang, Min Yang, Chao Yu, Xiaomei Zhang

**Affiliations:** 1 Department of Preparation Center, Chongqing Traditional Chinese Medicine Hospital, Chongqing, China; 2 College of Pharmacy, Chongqing Medical University, Chongqing, China; 3 Institute of Medicinal Chemistry, Chongqing Academy of Chinese Materia Medica, Chongqing, China; 4 Department of Pathology, The First Affiliated Hospital of Chongqing University of Chinese Medicine, Chongqing, China

**Keywords:** pharmacology, phytochemistry, *Rhododendron molle*, toxicology, traditional uses

## Abstract

*Rhododendron molle*, a member of the Ericaceae family, is a well-known medicinal plant in traditional Chinese medicine and ethnomedicine known as Yangzhizhu. As an important botanical drug, *R. molle* is characterized by “efficacy-toxicity duality”. This review aims to provide a comprehensive and up-to-date overview of *R. molle*, encompassing its traditional medicinal uses, clinical applications, phytochemistry, pharmacology, toxicology, pharmacokinetics, and quality control in recent years. Information on *R. molle* was collected from scientific journals, classical books on traditional Chinese herbal medicine, and reports through library and electronic searches (PubMed, Google Scholar, Web of Science, CNKI, and other authoritative databases. *R. molle* is distributed in regions of China, and is used for treating pain, rheumatoid arthritis, cardiovascular disease, and gastrointestinal disorders. Approximately 349 chemical metabolites have been identified from *R. molle*, including diterpenoids, triterpenoids, flavonoids, lignans, and other metabolites. Among these metabolites, diterpenoids are recognized as the main bioactive metabolites, showing significant analgesic, anti-inflammatory, immunomodulatory, and cardiovascular effects. However, it is important to note that *R. molle* exhibits neurotoxicity, cardiotoxicity, hepatotoxicity, with its toxicity being linked to its primary diterpenoids. Rhodojaponin II and rhodojaponin III are generally selected as indicators for the quantitative determination of *R. molle*. Therefore, *R. molle* represents a valuable yet highly toxic botanical drug, while its traditional uses are supported by modern pharmacological evidence, further research is needed to clarify the mechanisms underlying its toxic effects and to establish scientific strategies for detoxification and quality control.

## 1 Introduction

*Rhododendron*, the largest genus of Ericaceae, consists of approximately 1,000 species that are widely distributed in Europe, Asia and North America, with about 600 species found in China (Tian, et al., 2019). Species of this genus are renowned for their showy flowers and are commonly used as ornamental plants in landscaping (Park et al., 2018). More importantly, *Rhododendron* species have a long history of use in traditional Chinese medicine (TCM) for the treatment of various diseases, including chronic bronchitis, cough, rheumatic arthralgia, osteomyelitis, malignant boils, abdominal pain, and nephritis (Liu et al., 2024). Interestingly, some *Rhododendron* species, such as *R. molle* (Blume) G. Don and *Rhododendron micranthum* Turcz. are known for being poisonous to humans and livestock (Cai et al., 2018; Sun et al., 2019). A large amount of evidence has revealed that grayanane-type diterpenoids are responsible for the toxic effects of poisonous species; thus, grayananes are also called “grayanotoxins” (Dong et al., 2014; Yang et al., 2022b). Mad honey, is a type of honey produced by bees that collect nectar from grayanotoxins-containing *Rhododendron* plants; it has medicinal value in the management of hypertension, diabetes, and gastrointestinal disorders, yet also carries health risks (Aryal, 2025). In addition to diterpenoids, *Rhododendron* plants produce a diverse array of secondary metabolites such as triterpenoids, flavonoids, lignans, phenolic acids, sesquiterpenoids, and monoterpenoids, many of which exhibit remarkable pharmacological activities, particularly prominent analgesic and anti-inflammatory effects (Mei et al., 2025; Zheng et al., 2024a).

*Rhododendron molle* (known as *Yangzhizhu*), a toxic species endemic to China, is widely distributed in the Yangtze River basin in southern China, spanning Jiangsu, Anhui, Zhejiang, Jiangxi, Fujian, Henan, Hubei, Hunan, Guangdong, Guangxi, Sichuan, Guizhou, and Yunnan provinces (Flora of China Editorial Committee, 2004). This plant holds a significant position in TCM, first documented in Shennong’s Herbal Classic (circa 25–220 CE) and serving as an important material in Ma Fei San, an anesthetic formula developed by Hua Tuo during the late Eastern Han Dynasty (184–220 CE) (Chen et al., 2023). According to ancient TCM classics and modern pharmaceutical references, the flowers (known as *Naoyanghua*), fruits (known as *Liuzhouzi*), roots, and leaves of *R. molle* are all medicinally utilized, mainly for the management of rheumatic arthralgia and various pain syndromes (Nanjing University of Chinese Medicine, 2006). Among these, the flower is the most frequently employed medicinal part and serves as a crucial raw material in some medicinal preparations, such as Liuwei Muxiang San and Shengfa Chaji. Nevertheless, the entire plant of *R. molle* is highly toxic, and its flower is listed as one of the ten highly toxic TCMs in the current Chinese Pharmacopoeia (Chinese Pharmacopoeia Commission, 2025). Due to its extreme toxicity and narrow therapeutic window (effective dose close to toxic dose), its clinical dosage is strictly restricted to 0.6–1.5 g. Modern studies have revealed that *R. molle* is rich in diverse secondary metabolites including diterpenoids, triterpenoids, flavonoids, and lignans (Cai et al., 2018; Liu et al., 2024). Among these, diterpenoid metabolites possess significant analgesic, anti-inflammatory, immunomodulatory, and antiviral activities (Mei et al., 2025; Zheng et al., 2024a; Zhou et al., 2017a), while also exerting neurotoxicity, cardiotoxicity, and hepatotoxicity (Dong et al., 2014; Ran et al., 2025; Yang et al., 2022b). Such an “efficacy-toxicity duality” of *R. molle* and its diterpenoid metabolites presents both challenges and opportunities for its clinical application and pharmaceutical development.

Extensive efforts have been made to characterize the metabolites and biological activities of *R. molle*, and some scientific articles have been published (Cai et al., 2018; Mei et al., 2025). While most research has centered on its phytochemistry and pharmacology, a comprehensive review integrating its clinical indications, pharmacokinetics, and quality control is lacking. Importantly, new developments in analytical technologies have led to the isolation of new metabolites and additional detailed research on their pharmacological and toxicological activities. (Guo et al., 2025; Ran et al., 2025; Wang et al., 2025; Zheng et al., 2024b). Therefore, this review aims to provide a comprehensive overview of *R. molle*, covering its traditional uses, clinical applications, phytochemistry, pharmacology, toxicology, pharmacokinetics, and quality control. It is expected to provide a crucial theoretical basis and valuable perspectives for future research, supporting the safe and effective application of *R. molle* in clinical practice.

## 2 Methodology

A comprehensive analysis was performed on publications addressing botanical characteristics, traditional uses, clinical applications, phytochemistry, pharmacology, toxicology, pharmacokinetics, and quality control. Literature search was conducted with the keywords “Rhododendri Mollis Flos,” “*R. molle*,” “Yangzhizhu,” from Web of Science, PubMed, CNKI, Wanfang Database, and Google Scholar. Manual reading was performed to eliminate duplicate literature works and irrelevant content in the database. The exclusion criteria were articles without full texts and irrelevant articles that fell outside the scope of this review. Based on classic Chinese medicine books and relevant literature collected from various databases mentioned above, all eligible studies were analyzed and summarized. A total of over 500 publications from 1976 to December 2025 were initially identified, among which articles focusing on plant diversity, ecology, physiology, and pathology of *R. molle* were excluded. Ultimately, 134 publications were cited in this review.

## 3 Botanical characterization

**FIGURE 1:**
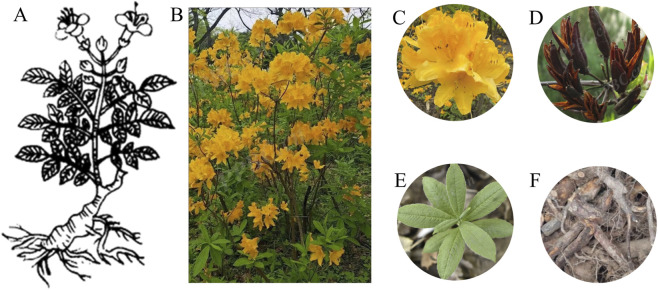
Morphological characteristics of *Rhododendron molle*. **(A)** the picture of *R. molle* in “Bencao Tujing”; **(B)** the whole plants of *R. molle*; **(C)** the flowers of *R. molle*; **(D)** the fruits of *R. molle*. **(E)** the leaves of *R. molle*; **(F)** the roots of *R. molle*.

*Rhododendron*, the largest genus in the Ericaceae family, comprises approximately 1,000 species widely distributed across Europe, Asia, and North America. According to the Flora of China (Flora of China Editorial Committee, 2004), there are 571 species of *Rhododendron* in China. Among them, *R. molle* stands out with its notable feature (yellow flowers), a trait that distinguishes it from many other species. *R. molle* was first documented with illustrations in the Bencao Tujing (Song Dynasty), which described its botanical characteristics (Figure 1A) as follows: “It grows in the mountain valleys of Taihang and Huaishan. It sprouts in spring, resembling Lycoris squamigera; its leaves are similar to those of *Carthamus tinctorius*; the stem reaches about three to four chi in height. It blooms in summer, with flowers resembling those of *Campsis grandiflora*, *Punica granatum*, or *Inula japonica*, and is pure yellow in color.”

The Flora of China (Flora of China Editorial Committee, 2004) provide a comprehensive botanical description of *R. molle*, including its morphology, distribution, and habitat. *R. molle* is a deciduous shrub, 0.5–2 m tall, with sparse, erect branches that are densely grayish-white pubescent and sparsely covered with coarse hairs when young. Its leaves are papery, oblong to oblong-lanceolate, 5–11 cm long and 1.5–3.5 cm wide, with an obtuse apex bearing a short mucro, a cuneate base, and ciliate margins. The adaxial surface is sparsely pubescent when young, while the abaxial surface is densely grayish-white pubescent with yellowish-brown setae along the prominently raised midvein; lateral veins are also conspicuous. Petioles are 2–6 mm long, pubescent, and sparsely setose. The inflorescence is a terminal racemose-umbel bearing up to 13 flowers, emerging before or concurrently with the leaves. Pedicels are 1–2.5 cm long, sparsely pubescent, and with scattered coarse hairs. Calyx lobes are small, crenate, and covered with fine hairs and bristle-like cilia. The corolla is broadly funnel-shaped, 4.5 cm long and 5–6 cm in diameter, yellow to golden-yellow with deep red spots internally; the corolla tube narrows toward the base, is cylindrical, 2.6 cm long, and externally pubescent. Corolla lobes number five, elliptic to ovate-oblong, 2.8 cm long, and pubescent externally. Stamens number five, unequal in length, not exceeding the corolla; filaments are flattened and pubescent below the middle. The ovary is conical, 4 mm long, densely grayish-white pubescent and sparsely setose; the style is up to 6 cm long and glabrous. The capsule is conical-oblong, 2.5–3.5 cm long, with five distinct longitudinal ribs, covered with fine hairs and sparse coarse hairs. Flowering occurred from March to May, and fruiting from July to August.

*R. molle* is widely distributed in the Yangtze River basin of southern China, across Jiangsu, Anhui, Zhejiang, Jiangxi, Fujian, Henan, Hubei, Hunan, Guangdong, Guangxi, Sichuan, Guizhou, and Yunnan. It inhabits grassy slopes, thickets in hilly areas, or mixed forests along mountain ridges, at elevations of approximately 1,000 m. This species is easily distinguishable within Ericaceae by its relatively large leaves, densely grayish-white pubescent and sparsely setose, and its large yellow to golden-yellow corolla. Whole plants of *R. molle* are shown in Figure 1B, with detailed images of the flowers, fruits, leaves, and roots provided in Figures 1C–F.

## 4 Traditional uses and clinical applications

In China, approximately 25 species of *Rhododendron* are used as traditional medicines or folk remedies to treat various diseases, of which *R. molle* has the longest medicinal use history. Medicinal records of *R. molle* can be traced back to the Han Dynasty. Shennong’s Herbal Classic recorded it as “Yangzhizhu,” without specifying the medicinal parts of the plant. The modern Dictionary of TCM (Nanjing University of Chinese Medicine, 2006) includes its flowers, fruits, and roots, which were used to treat rheumatic arthralgia and various types of pain. Currently, the flowers of *R. molle* (named Naoyanghua) are officially included in the Chinese Pharmacopoeia (2025 edition) and represent the most frequently used medicinal part. Additionally, recent studies have also identified that the leaves exhibit notable anti-inflammatory and analgesic activities, indicating potential for further development (Luo et al., 2023a; Luo et al., 2023b).

*R. molle* is typically applied in clinical practice within botanical formulas. Classical texts documenting traditional formulas are listed in Table 1, showing its use for treating traumatic injuries, cutaneous lesions, wind-syndromes, painful obstruction (Bi-syndrome), and epidemic afflictions. Today, it is processed into multiple preparation forms for clinical application, with its therapeutic uses for various diseases summarized in Table 2.

**TABLE 1:** The traditional uses of the *Rhododendron molle* in ancient books.

Preparation name	Main compositions	Traditional uses	References
Mafei San	*R. molle* (flower), *Angelica sinensis* (root), *Acorus tatarinowii* (root and stem), *et al.*	Anesthesia and analgesia	Hua Tuo Shen Fang (《华佗神方》) Han Dynasty
Jiezhi Wan	*R. molle* (flower, wine-steamed), *Schizonepeta tenuifolia* (aboveground part), *Angelica dahurica* (root), *et al.*	Cutaneous lesions and various wind-syndromes	Jiewei Yuansuo (《解围元薮》) Ming Dynasty
Sanling Wan	*R. molle* (flower), *Sophora flavescens* (root), *Schizonepeta tenuifolia* (aboveground part), *et al.*	Various wind-syndromes	Jiewei Yuansuo (《解围元薮》) Ming Dynasty
Jiuku Huisheng Dan	*R. molle* (flower), *Boswellia carterii* (resin),*Commiphora myrrha* (resin), *et al.*	Painful obstruction (Bi-syndrome)	Jiewei Yuansuo (《解围元薮》) Ming Dynasty
Lafan Wan	*R. molle* (flower, wine-processed), *Aconitum kusnezoffii* (root)	Various wind-syndromes	Jiewei Yuansuo (《解围元薮》) Ming Dynasty
Baji Gao	*R. molle* (flower), *Croton tiglium* (fruit), *Bletilla striata* (root and stem), *et al.*	Cutaneous lesions	Ji Ming Lu (《鸡鸣录》) Qing Dynasty
Ciyun San	*R. molle* (flower), *Aconitum carmichaelii* (root),*Eupolyphaga sinensis*, *et al.*	Traumatic injuries, and cutaneous lesions	Waike Huizhuan (《伤科汇纂》) Qing Dynasty
Damen Yuanding	*R. molle* (flower), *Aconitum carmichaelii* (root), *Angelica dahurica* (root), *et al.*	Various wind-syndromes	Chuan Ya Bu (《串雅补》) Qing Dynasty
Huxin Qili San	*R. molle* (fruit, wine-processed), *Platycodon grandiflorum* (root), *Angelica sinensis* (root), *et al.*	Traumatic injuries	Huiyi Tang Jingyan Fang (《蕙怡堂经验方》) Qing Dynasty
Jiling Jiegu Gao	*R. molle* (flower), *Rehmannia glutinosa* (root), *Rheum palmatum* (root), *et al.*	Traumatic injuries	Zhongfu Tang Fang (《种福堂方》) Qing Dynasty
Diyi Lingbao Dan	*R. molle* (flower), *Bufo bufo gargarizans* (secretion), *Zingiber officinale* (root), *et al.*	Epidemic afflictions, cutaneous lesions	Jingyan Qifang (《经验奇方》) Qing Dynasty
Jutong Wuyou San	*R. molle* (flower, vinegar-processed), *Atractylodes lancea* (root and stem), *Pinellia ternate* (stem), *et al.*	Anesthesia and analgesi, painful obstruction (Bi-syndrome)	Waike Dacheng (《外科大成》) Qing Dynasty
Qionghua Gao	*R. molle* (root), *Acanthopanax gracilistylus* (root bark), *Angelica sinensis* (root), *et al.*	Cutaneous lesions	Waike Dacheng (《外科大成》) Qing Dynasty
Qiongsu Gao	*R. molle* (flower), *Bufo bufo gargarizans*, *Pinellia ternate* (stem), *et al.*	Traumatic injuries, and cutaneous lesions	Waike Dacheng (《外科大成》) Qing Dynasty
Qiongye Gao	*R. molle* (flower), *Angelica sinensis*(root), *Carthamus tinctorius* (flower)*, et al.*	Traumatic injuries	Yizong Jinjian (《医宗金鉴》) Qing Dynasty
Qiongye San	*R. molle* (flower)	Traumatic injuries	Yizong Jinjian (《医宗金鉴》) Qing Dynasty

**TABLE 2:** The clinical application of the *Rhododendron molle* in modern books.

Preparation name	Main compositions	Clinical application	References
Liuwei Muxiang San	*R. molle* (flower), *Aucklandia lappa* (root), *Gardenia jasminoides* (fruit), *et al.*	Stomach fullness and pain	Chinese Pharmacopoeia (2025 edition)
Shengfa Chaji	*R. molle* (flower), Psoralea corylifolia (fruit), *Zingiber officinale* (root)	Alopecia areata	Chinese Pharmacopoeia (2025 edition)
Hu’richa Liuwei Wan	*R. molle* (flower), erminalia chebula (fruit), *Aucklandia lappa* (root), *et al.*	Headache	Ministry of Health Drug Standards (Mongolian volume)
Fengshi Er’shiwu Wei Wan	*R. molle* (flower), *G. jasminoides* (fruit), *Sophora flavescens (root), et al.*	Migraine, arthritis, and rheumatoid	Ministry of Health Drug Standards (Mongolian volume)
Tongqiao San	*R. molle* (flower), *Moschus berezovskii* (secretion), *Juncus effusus*, *et al.*	Heatstroke, fainting	Ministry of Health Drug Standards (volume 4)
Guanjie Jietong Gao	*R. molle* (flower), *Angelica dahurica* (root), *Asarum heterotropoides* (root and stem), *et al.*	Arthralgia, neuralgia, back pain, muscle soreness, and sprain	Ministry of Health Drug Standards (volume 10)
Zhitong Shui	*R. molle* (flower), *Stephania tetrandra* (root), *Corydalis yanhusuo* (root), *et al.*	Headache, toothache, arthralgia, and neuralgia	Ministry of Health Drug Standards (volume 2)
Wolong San	*R. molle* (flower), *Moschus berezovskii* (secretion), *Bufo gargarizans* (secretion), *et al.*	Heatstroke, fainting, and infantile convulsion	Ministry of Health Drug Standards (volume 1)

### 4.1 Anesthesia and analgesia

Historically, *R. molle* flowers served as the principal material in “Mafei San,” an early anesthetic formula attributed to Hua Tuo during the late Eastern Han Dynasty (Chen et al., 2023). Clinical practice has shown that the combination of *R. molle* flower injection and *Datura metel* injection produced a synergistic anesthetic effect. Additionally, some modern preparations exploited its anesthetic and analgesic properties, including “Zhitong Shui” and “Huricha Liuwei Wan,” which were used for headaches, toothaches, and neuralgias. For safety reasons, the clinical use of *R. molle* injections has been halted, with their current research value lying primarily in the discovery of lead metabolites.

### 4.2 Rheumatoid arthritis

Rheumatoid arthritis (RA) is a chronic autoimmune disease affecting about 0.5–1% of the population worldwide, mainly characterized by chronic, symmetrical, synovial arthritis and extraarticular lesions (Zhu et al., 2022). RA is referred to in TCM as “Bi-syndrome,” a condition believed to result from wind, cold, and dampness. *R. molle* has a long history of use in “Bi-syndrome” treatment, dating back to its first documentation in Shennong’s Herbal Classic. The Chinese Pharmacopoeia (2025 edition) continues to record its therapeutic effects, noting its ability to “dispel wind, eliminate dampness, dissipate stasis, and alleviate pain.” A 1989 study reported that applying fresh *R. molle* flower paste on affected joints, combined with oral administration of a decoction prepared from dried *R. molle* flowers, significantly reduced joint swelling and pain, decreased erythrocyte sedimentation rate, and achieved an overall efficacy rate of 91.1% in RA patients. In another clinical evaluation, tablets derived from *R. molle* roots (0.5 g per tablet) exhibited a total effective rate of 87%. Additionally, a composite pill of *R. molle* root extracts with *ginseng* and *pangolin*, at daily doses of 4.5–7.5 g, significantly alleviated symptoms in RA patients. Moreover, a Mongolian medicinal preparation containing *R. molle* flowers (Fengshi Er’shi Wuwei Wan) demonstrated a total effective rate of 93.3% in the clinical treatment of RA (Jun et al., 2020). Although the existing clinical data are encouraging, most of these studies were published in the 1980s and 1990s and lack the support of rigorous, large-scale randomized controlled trials (RCTs). Therefore, future research should prioritize well-designed clinical studies to definitively establish its efficacy.

### 4.3 Insecticides

Historically, *R. molle* flowers were documented for oral use against intestinal parasites such as roundworms and tapeworms. However, due to substantial risks associated with internal use, this method has largely been abandoned. In folk practices, crushed flowers were soaked to prepare liquid extracts used as agricultural sprays, effectively eradicating aphids and borers from crops. Additionally, *R. molle* extracts demonstrated insecticidal activity against various species, including *Reticulitermes angusticollis*, *Locusta migratoria manilensis*, and *Schistosoma japonicum* (Cheng et al., 2011). Consequently, its external application as an insecticide has emerged as the primary direction for its utilization.

### 4.4 Skin diseases

*R. molle* flowers has a long history of topical application in the treatment of dermatological conditions, serving as an essential material in traditional formulations such as “Baji Gao” “Qionghua Gao” and “Qiongsu Gao” for managing various skin disorders. Its efficacy against “stubborn lichen” (a category of refractory dermatoses) was officially recognized in the Chinese Pharmacopoeia (2025 edition). Furthermore, the modern proprietary Chinese medicine preparation “Shengfa Chaji,” which contains *R. molle* flowers as a raw material, has been clinically used for the treatment of alopecia areata, thereby expanding its therapeutic scope in the field of dermatology.

### 4.5 Cardiovascular diseases

The cardioprotective properties of *R. molle* represented a notable discovery in modern pharmacology, as no such applications were documented in classical medical texts. Rhodojaponin III (RJ-III, **16)**, a metabolite extracted from *R. molle*, was formulated into tablets and injections for managing various supraventricular tachyarrhythmias, including paroxysmal supraventricular tachycardia, multifocal atrial premature contractions, rapid atrial fibrillation, and sinus tachycardia, as well as for hypertension (Chinese Pharmacopoeia Commission, 1977). Studies demonstrated that RJ-III (**16**) injection was effective in 80.95% of patients with severe hypertension, exhibiting potent and rapid-onset antihypertensive effects. In cases of pregnancy-induced hypertension, RJ-III (**16**) injection reduced systolic and diastolic blood pressure by 29.6% and 32.3%, respectively, achieving a marked effectiveness rate of 96%, with no significant impact on fetal heart rate. However, its clinical application was ultimately limited by a narrow therapeutic window, posing significant risks of severe adverse effects, including profound hypotension, shock, and cardiac arrest. Consequently, it is no longer used as a conventional cardiovascular therapy in clinical practice.

### 4.6 Other application

*R. molle* has also been documented for use in the treatment of orthopedic and gastrointestinal disorders. Traditionally, formulations such as “Ciyun San” and “Huxin Qili San”, which contain *R. molle* flowers, were employed for managing traumatic injuries such as bruises and contusions, leveraging its properties of dispersing blood stasis and alleviating pain. Meanwhile, “Liuwei Muxiang San,” composed of *R. molle* flowers, *Piper longum*, *Amomum kravanh*, and other medicinal botanical drugs, was used for the treatment of stomach pain, reflecting the therapeutic value of *R. molle* in digestive system disorders (Dhondrup et al., 2023).

## 5 Phytochemistry

**FIGURE 2:**
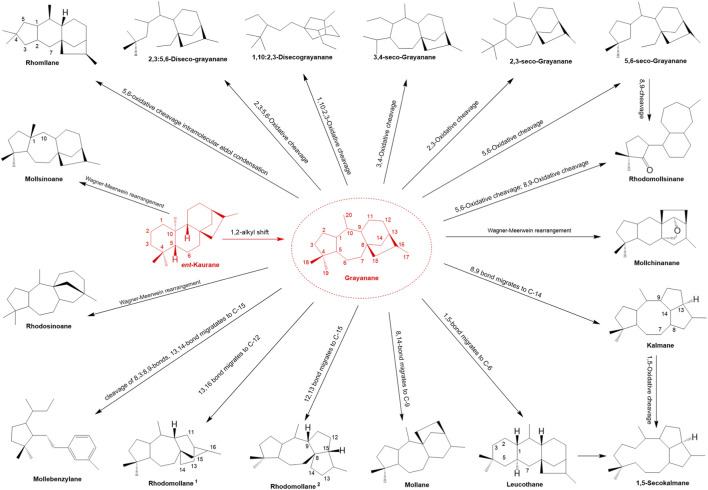
Classifications of diterpenoids from *Rhododendron molle* and proposed biogenetic pathways.

**FIGURE 3:**
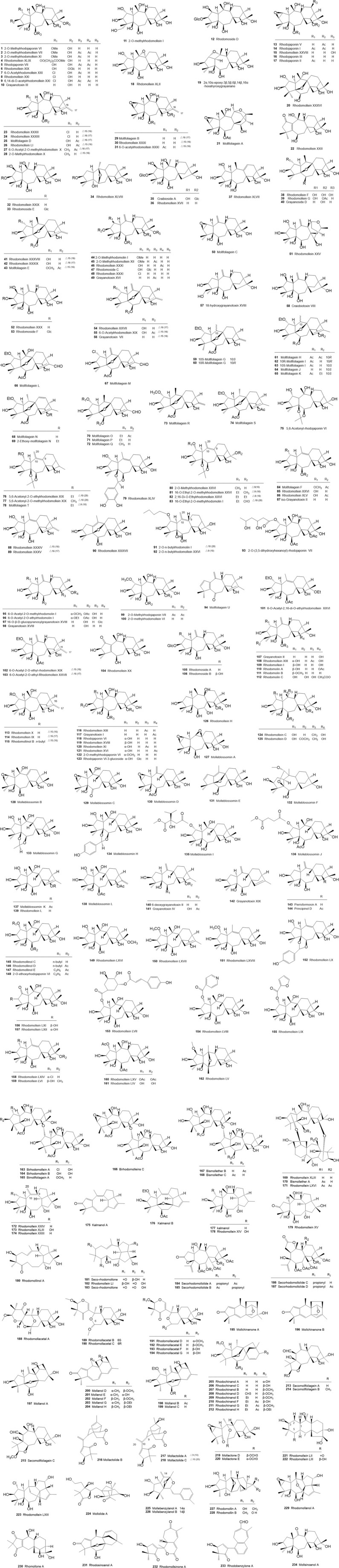
Structures of diterpenoids isolated and identified from *Rhododendron molle*.

**FIGURE 4:**
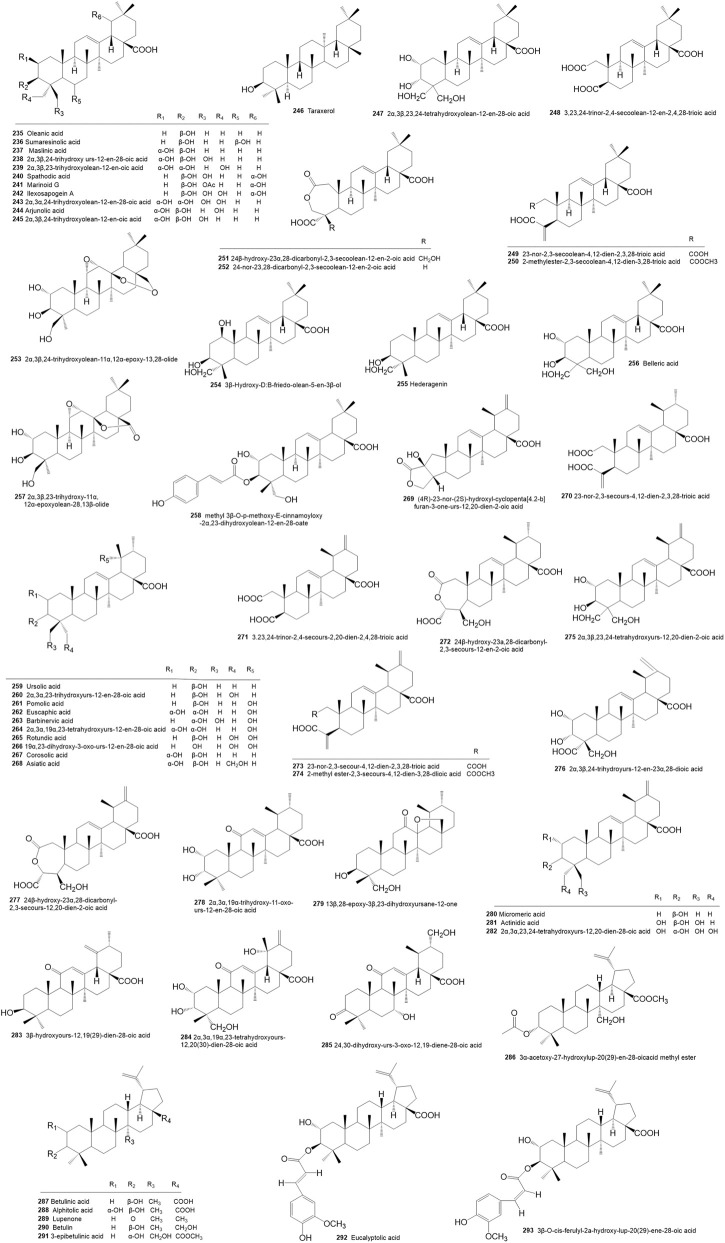
Structures of triterpenoids isolated and identified from *Rhododendron molle*.

**FIGURE 5:**
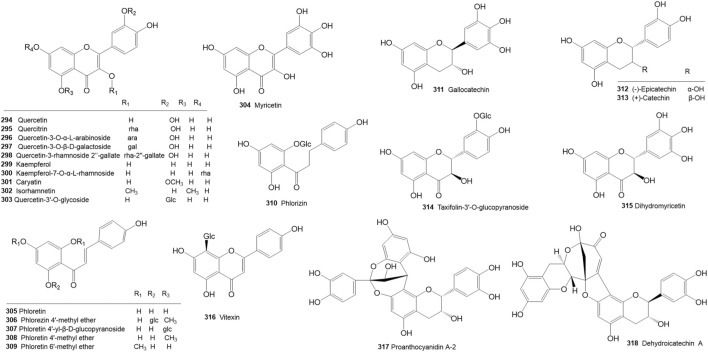
Structures of flavonoids isolated and identified from *Rhododendron molle*.

**FIGURE 6:**
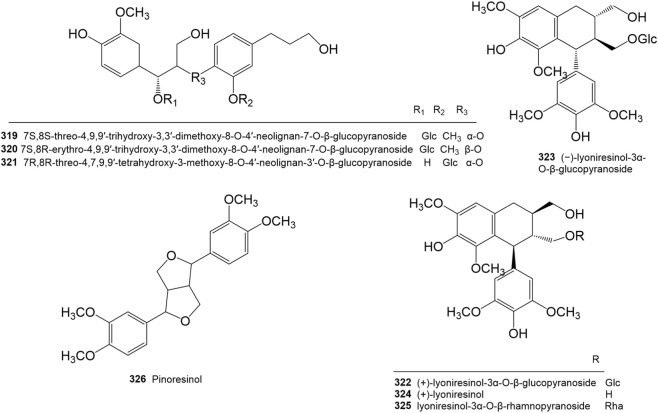
Structures of lignans isolated and identified from *Rhododendron molle*.

To date, a total of 344 metabolites have been reported in *R. molle*, including 234 diterpenoids, 53 triterpenoids, 26 flavonoids, 8 lignans, and 23 others (Tables 3–7). Chemdraw software was used to depict the chemical structures of these metabolites (Figures 2–6). The characteristic metabolites are highlighted in the following sections.

**TABLE 3:** Diterpenoids isolated and identified from *Rhododendron molle*.

No.	Classifications	Name	Plant part	References
1	Grayanane	2-O-Methylrhodojaponin VI	Flowers, leaves	Zhou et al. (2018b), Zheng et al. (2024b)
2	Grayanane	2-O-Methylrhodomollein VII	Leaves	Zhou et al. (2018b)
3	Grayanane	2-O-Methylrhodomollein XI	Leaves, flowers	Li et al. (2020), Zheng et al. (2024b), Zhou et al. (2018b), Zhou et al. (2014)
4	Grayanane	Rhodomollein XLIX	Flowers	Li et al. (2020)
5	Grayanane	Rhodojaponin VII	Leaves, flowers	Zheng et al. (2024a)
6	Grayanane	Rhodomollein XIX	Roots, flowers, fruits	Li et al. (2015), Zhang (2012), Zheng et al. (2024a)
7	Grayanane	6-O-Acetylrhodomollein XXI	Leaves, flowers, fruits	Zheng et al. (2024a), Zhou et al. (2018b)
8	Grayanane	Rhodomollein XXI	Flowers, fruits	Zhou et al. (2014)
9	Grayanane	6,14-di-O-acetylrhodomollein XXI	Flowers	Zhou et al. (2014)
10	Grayanane	Grayanotoxin III	Roots, flowers	Li et al. (2015), Zhang (2012)
11	Grayanane	Rhodomollein XLVI	Flowers	Li et al. (2020)
12	Grayanane	Rhodomoside D	Roots	Li et al. (2015)
13	Grayanane	Rhodojaponin V	Leaves, flowers	Zhou et al. (2018b)
14	Grayanane	Rhodojaponin I	Leaves, flowers	Zheng et al. (2024a), Zhou et al. (2014)
15	Grayanane	Rhodomollein XXVIII	Roots	Li et al. (2015)
16	Grayanane	Rhodojaponin III	Leaves, flowers, roots	Li et al. (2000), Li et al. (2015), Zheng et al. (2024a), Zhou et al. (2014)
17	Grayanane	Rhodojaponin II	Leaves, flowers	Song et al. (2025), Zheng et al. (2024a), Zhou et al. (2014)
18	Grayanane	Rhodomollein XLII	Fruits	Li et al. (2018)
19	Grayanane	2α,10α-epoxy-3β,5β,6β,14β,16α-hexahydroxygrayanane	Flowers	Chen et al. (2013)
20	Grayanane	Rhodomollein XXXVI	Fruits	Li et al. (2018)
21	Grayanane	Mollfoliagein A	Leaves	Zhou et al., 2018b; Zhou (2018)
22	Grayanane	Rhodomollein XXII	Flowers	Zhou et al. (2014)
23	Grayanane	Rhodomollein XXXII	Fruits	Li et al. (2018)
24	Grayanane	Rhodomollein XXXIII	Fruits	Li et al. (2018)
25	Grayanane	Mollfoliagein D	Leaves	Zhou et al. (2018b)
26	Grayanane	Rhodomollein LI	Flowers	Li et al. (2020)
27	Grayanane	6-O-Acetyl-2-O-methylrhodomollein X	Leaves	Zhou (2018)
28	Grayanane	2-O-Methylrhodomollein X	Leaves	Zhou (2018)
29	GrayananeGrayanane	Mollfoliagein B	Leaves, flowers, fruits	Zheng et al. (2024a), Zhou et al. (2018b)
30	Grayanane	Rhodomollein XXXI	Fruits, flowers, leaves	Li et al. (2020), Zheng et al. (2024a), Zhou, 2018
31	Grayanane	6-O-Acetyl-rhodomollein XXXI	Leaves	Zhou et al., 2018b; Zhou (2018)
32	Grayanane	Rhodomollein XXIX	Roots	Li et al. (2015)
33	Grayanane	Rhodomoside E	Roots	Li et al. (2015)
34	Grayanane	Rhodomollein XLVIII	Flowers	Li et al. (2020)
35	Grayanane	Craiobioside A	Roots	Li et al. (2015)
36	Grayanane	Rhodomollein XVII	Fruits	Li et al. (2000)
37	Grayanane	Rhodomollein XLVII	Flowers	Li et al. (2020)
38	Grayanane	Rhodomollein F	Flowers, fruits	Zhang Z. R. (2012)
39	Grayanane	Rhodomollein G	Flowers	Zhang Z. R. (2012)
40	Grayanane	Grayanoside D	Flowers	Zhang Z. R. (2012)
41	Grayanane	Rhodomollein XXXVIII	Fruits	Li et al. (2018)
42	Grayanane	Rhodomollein XXXIX	Fruits	Li et al. (2018)
43	Grayanane	Mollfoliagein E	Leaves	Zhou et al. (2018b)
44	Grayanane	2-O-Methylrhodomolin I	Leaves	Zhou et al. (2018b)
45	Grayanane	2-O-Methylrhodomollein XII	Leaves	Zhou et al. (2018b)
46	Grayanane	Rhodomolin I	Leaves, roots	Zheng et al. (2024a), Zhou et al. (2018b)
47	Grayanane	Rhodomoside C	Roots	Li et al. (2015)
48	Grayanane	Rhodomollein XXXI	Leaves	Li et al. (2020)
49	Grayanane	Grayanotoxin XVI	Leaves, flowers	Zheng et al. (2024a), Zhou et al. (2018b)
50	Grayanane	Mollfoliagein C/Rhodomollein XL	Leaves, flowers	Zheng et al. (2024a), Zhou et al. (2018b)
51	Grayanane	Rhodomollein XXV	Fruits	Li et al. (2013)
52	Grayanane	Rhodomollein XXX	roots, flowers	Zheng et al. (2024a)
53	Grayanane	Rhodomoside F/1β-Rhodomoside B	Roots	Li et al. (2015)
54	Grayanane	Rhodomollein XXVII	Roots	Zheng et al. (2024a)
55	Grayanane	6-O-Acetylrhodomollein XIX	Leaves	Zhou et al. (2018b)
56	Grayanane	Grayanotoxin VII	Fruits	Li et al. (2018)
57	Grayanane	18-Hydroxygrayanotoxin XVIII	Leaves	Zhou et al. (2018b)
58	Grayanane	Craiobiotoxin VIII	Roots	Li et al. (2015)
59	Grayanane	10S-Mollfoliagein G	Leaves	Zhou (2018)
60	Grayanane	10R-Mollfoliagein G	Leaves	Zhou (2018)
61	Grayanane	Mollfoliagein H	Leaves	Zhou (2018)
62	Grayanane	10R-Mollfoliagein I	Leaves	Zhou (2018)
63	Grayanane	10S-Mollfoliagein I	Leaves	Zhou (2018)
64	Grayanane	Mollfoliagein J	Leaves	Zhou (2018)
65	Grayanane	Mollfoliagein K	Leaves	Zhou (2018)
66	Grayanane	Mollfoliagein L	Leaves	Zhou (2018)
67	Grayanane	Mollfoliagein M	Leaves	Zhou (2018)
68	Grayanane	Mollfoliagein N	Leaves	Zhou (2018)
69	Grayanane	2-Ethoxy-mollfoliagein N	Leaves	Zhou (2018)
70	Grayanane	Mollfoliagein O	Leaves	Zhou (2018)
71	Grayanane	Mollfoliagein P	Leaves	Zhou (2018)
72	Grayanane	Mollfoliagein Q	Leaves	Zhou (2018)
73	Grayanane	Mollfoliagein R	Leaves	Zhou (2018)
74	Grayanane	Mollfoliagein S	Leaves	Zhou (2018)
75	Grayanane	5,6-Acetonyl-rhodojaponin VI	Leaves	Zhou (2018)
76	Grayanane	5,6-Acetonyl-2-O-ethylrhodomollein XIX	Leaves	Zhou (2018)
77	Grayanane	5,6-Acetonyl-2-O-methylrhodomollein XIX	Leaves	Zhou (2018)
78	Grayanane	Mollfoliagein T	Leaves	Zhou (2018)
79	Grayanane	Rhodomollein XLIV	Flowers	Li et al. (2020)
80	Grayanane	2-O-Methylrhodomollein XXVI	Leaves	Zhou (2018)
81	Grayanane	16-O-Ethyl-2-O-methylrhodomollein XXVI	Leaves	Zhou (2018)
82	Grayanane	2,16-Di-O-Ethylrhodomollein XXVI	Leaves	Zhou (2018)
83	Grayanane	16-O-Ethyl-2-O-methylrhodomolin I	Leaves	Zhou (2018)
84	Grayanane	Mollfoliagein F	Leaves	Zhou et al. (2018b)
85	Grayanane	Rhodomollein XXVI	roots, flowers	Zheng et al. (2024a)
86	Grayanane	Rhodomollein XLV	Flowers	Li et al. (2020)
87	Grayanane	*iso*-Grayanotoxin II	Fruits, flowers	Li et al. (2018), Zheng et al. (2024a)
88	Grayanane	Rhodomollein XXXIV	Fruits	Li et al. (2018)
89	Grayanane	Rhodomollein XXXV	Fruits	Li et al. (2018)
90	Grayanane	Rhodomollein XXXVII	Fruits	Li et al. (2018)
91	Grayanane	2-O-n-butylrhodomolin I	Leaves	Zhou (2018)
92	Grayanane	2-O-n-butylrhodomollein XXVI	Leaves	Zhou (2018)
93	Grayanane	2-O-(3,5-dihydroxyhexanoyl)-rhodojaponin VII	Leaves	Zhou (2018)
94	Grayanane	Mollfoliagein U	Leaves	Zhou (2018)
95	Grayanane	6-O-Acetyl-2-O-methylrhodomolin I	Leaves	Zhou (2018)
96	Grayanane	6-O-Acetyl-2-O-ethylrhodomolin I	Leaves	Zhou (2018)
97	Grayanane	16-O-β-D-glucopyranosyl-grayanotoxin XVIII	Leaves	Zhou (2018)
98	Grayanane	Grayanotoxin XVIII	Leaves	Zhou et al. (2018b)
99	Grayanane	2-O-Methylrhodojaponin VII	Flowers, Leaves	Zheng et al., 2024b; Zhou (2018)
100	Grayanane	2-O-Methylrhodomollein VI	Leaves	Zhou et al., 2018b; Zhou (2018)
101	Grayanane	6-O-Acetyl-2,16-di-O-ethylrhodomollein XXVI	Leaves	Zhou (2018)
102	Grayanane	6-O-Acetyl-2-O-ethyl-rhodomollein XIX	Leaves	Zhou (2018)
103	Grayanane	6-O-Acetyl-2-O-ethyl-Rhodomollein XXVII	Leaves	Zhou (2018)
104	Grayanane	Rhodomollein XX	Fruits	Li et al. (2013)
105	Grayanane	Rhodomoside A	Roots	Chen et al. (2013)
106	Grayanane	Rhodomoside B	Roots	Li et al. (2015)
107	Grayanane	Grayanotoxin II	Leaves, flowers, roots	Zhou et al. (2018b), Zheng et al. (2024a)
108	Grayanane	Rhodomollein XII	Leaves, flowers	Song et al. (2025), Zheng et al. (2024a)
109	Grayanane	Rhodomollein I	Leaves, flowers, roots	Li et al. (2015), Zhou et al. (2018b), Zhou et al. (2014)
110	Grayanane	Rhodomolin A	Flowers	Zhong et al. (2004)
111	Grayanane	Rhodomolin B	Flowers	Zhong et al. (2004)
112	Grayanane	Rhodomolin C	Flowers	Zhong et al. (2004)
113	Grayanane	Rhodomollein X	Flowers	Song et al. (2025), Zheng et al. (2024a), Zheng et al. (2024b)
114	Grayanane	Rhodomollein IX	Flowers	Zhang Z. R. (2012)
115	Grayanane	Rhodomollinol B	Flowers	Zheng et al. (2024b)
116	Grayanane	Rhodomollein XIII	Leaves, flowers	Song et al. (2025), Zhou et al. (2018b)
117	Grayanane	Grayanotoxin I	Flowers	Zhong et al. (2004)
118	Grayanane	Rhodojaponin VI	Leaves, flowers, roots	Li et al. (2000), Li et al. (2015), Zheng et al. (2024a)
119	Grayanane	Rhodomollein XVIII	Flowers, fruits	Li et al. (2000)
120	Grayanane	Rhodomollein XI/Rhodomollein III	Leaves, flowers	Song et al. (2025), Li et al. (2020), Zheng et al. (2024a)
121	Grayanane	Rhodomollein XVI	Flowers, fruits	Li et al. (2000), Zhang Z. R. (2012)
122	Grayanane	2-O-Methylrhodojaponin VI	Flowers	Zheng et al. (2024b)
123	Grayanane	Rhodojaponin VI-3-glucoside	Roots	Li et al. (2015)
124	Grayanane	Rhodomollein C	Flowers	Zhang Z. R. (2012)
125	Grayanane	Rhodomollein D	Flowers	Zhang Z. R. (2012)
126	Grayanane	Rhodomollein H	Flowers	Zhang Z. R. (2012)
127	Grayanane	Molleblossomin A	Flowers	Zheng et al. (2024a)
128	Grayanane	Molleblossomin B	Flowers	Zheng et al. (2024a)
129	Grayanane	Molleblossomin C	Flowers	Zheng et al. (2024a)
130	Grayanane	Molleblossomin D	Flowers	Zheng et al. (2024a)
131	Grayanane	Molleblossomin E	Flowers	Zheng et al. (2024a)
132	Grayanane	Molleblossomin F	Flowers	Zheng et al. (2024a)
133	Grayanane	Molleblossomin G	Flowers	Zheng et al. (2024a)
134	Grayanane	Molleblossomin H	Flowers	Zheng et al. (2024a)
135	Grayanane	Molleblossomin I	Flowers	Zheng et al. (2024a)
136	Grayanane	Molleblossomin J	Flowers	Zheng et al. (2024a)
137	Grayanane	Molleblossomin K	Flowers	Zheng et al. (2024a)
138	Grayanane	Molleblossomin L	Flowers	Zheng et al. (2024a)
139	Grayanane	Rhodomollein L	Flowers	Song et al. (2025) Li et al. (2020), Zheng et al. (2024a)
140	Grayanane	6-Deoxygrayanotoxin II	Flowers	Zheng et al. (2024a)
141	Grayanane	Grayanotoxin IV	Flowers	Zheng et al. (2024a)
142	Grayanane	Grayanotoxin XIX	Flowers	Zheng et al. (2024a)
143	Grayanane	Pierisformosin A/1-epi-grayanotoxin XVIII	Flowers, Leaves	Zheng et al. (2024a), Zheng et al. (2019)
144	Grayanane	1-epi-grayanotoxin II/Principinol D	Flowers, Leaves	Zhang et al. (2015), Zheng et al. (2024a)
145	Grayanane	Rhodomollinol C	Flowers	Zheng et al. (2024b)
146	Grayanane	Rhodomollinol D	Flowers	Zheng et al. (2024b)
147	Grayanane	Rhodomollinol E	Flowers	Zheng et al. (2024b)
148	Grayanane	2-O-ethoxyrhodojaponin VI	Flowers	Zheng et al. (2024b)
149	Grayanane	Rhodomollein LXVI	Flowers	Wang et al. (2025)
150	Grayanane	Rhodomollein LXVII	Flowers	Wang et al. (2025)
151	Grayanane	Rhodomollein LXVIII	Flowers	Wang et al. (2025)
152	Grayanane	Rhodomollein LX	Flowers	Song et al. (2025)
153	Grayanane	Rhodomollein LVII	Flowers	Song et al. (2025)
154	Grayanane	Rhodomollein LVIII	Flowers	Song et al. (2025)
155	Grayanane	Rhodomollein LIX	Flowers	Song et al. (2025)
156	Grayanane	Rhodomollein LXI	Flowers	Song et al. (2025)
157	Grayanane	Rhodomollein LXII	Flowers	Song et al. (2025)
158	Grayanane	Rhodomollein LXIV	Flowers	Song et al. (2025)
159	Grayanane	Rhodomollein LVI	Fruits	Zhu et al. (2024)
160	Grayanane	Rhodomollein LXV	Flowers	Song et al. (2025)
161	Grayanane	Rhodomollein LIV	Fruits	Zhu et al. (2024)
162	Grayanane	Rhodomollein LV	Fruits	Zhu et al. (2024)
163	dimeric diterpenoids	Birhodomollein A	Flowers	Shuai et al. (2017)
164	dimeric diterpenoids	Birhodomollein B	Flowers	Shuai et al. (2017)
165	dimeric diterpenoids	Bimollfoliagein A	Leaves	Zhou et al. (2018b)
166	dimeric diterpenoids	Birhodomollein C	Flowers	Shuai et al. (2017)
167	dimeric diterpenoids	Bismollether B	Flowers	Huang et al. (2022)
168	dimeric diterpenoids	Bismollether C	Flowers	Huang et al. (2022)
169	dimeric diterpenoids	Rhodomollein XLIII	Flowers, fruits	Li et al. (2020), Song et al. (2025)
170	dimeric diterpenoids	Bismollether A	Flowers	Huang et al. (2022)
171	dimeric diterpenoids	Rhodomollein LXVI	Flowers	Song et al. (2025)
172	kalmane	Rhodomollein XXIV	Flowers	Zhou et al. (2014)
173	kalmane	Rhodomollein XLIII	Flowers, fruits	Li et al. (2018)
174	kalmane	Rhodomollein XXIII	Flowers	Zheng et al. (2024b), Zhou et al. (2014)
175	kalmane	Kalmanol A	Leaves	Zhou (2018)
176	kalmane	Kalmanol B	Leaves	Zhou (2018)
177	kalmane	kalmanol	Flowers	Li et al. (2020)
178	kalmane	Rhodomollein XIV	Flowers	Zhou et al. (2014)
179	kalmane	Rhodomollein XV	Flowers	Zhou et al. (2014)
180	1,5-secokalmane	Rhodomollinol A	Flowers	Zheng et al. (2024b)
181	1,5-secokalmane	Seco-rhodomollone	Flowers	Li et al. (2020), Zheng et al. (2024b)
182	1,5-secokalmane	Rhodomollein LI	Flowers	Li et al. (2020)
183	1,5-secokalmane	Seco-rhodomollone	Flowers	Li et al. (2020)
184	3,4-secograyanane	Secorhodomollolide A	Flowers	Wang et al. (2010)
185	3,4-secograyanane	Secorhodomollolide B	Flowers	Wang et al. (2010)
186	3,4-secograyanane	Secorhodomollolide C	Flowers	Wang et al. (2010)
187	3,4-secograyanane	Secorhodomollolide D	Flowers	Wang et al. (2010)
188	2,3:5,6-diseco-grayanane	Rhodomollacetal A	Leaves	Zhou et al. (2017a)
189	2,3:5,6-diseco-grayanane	Rhodomollacetal B	Leaves	Zhou et al., 2017a; Zhou (2018)
190	2,3:5,6-diseco-grayanane	Rhodomollacetal C	Leaves	Zhou et al. (2017a)
191	2,3:5,6-diseco-grayanane	Rhodomollacetal D	Leaves	Zhou (2018)
192	2,3:5,6-diseco-grayanane	Rhodomollacetal E	Leaves	Zhou (2018)
193	2,3:5,6-diseco-grayanane	Rhodomollacetal F	Leaves	Zhou (2018)
194	2,3:5,6-diseco-grayanane	Rhodomollacetal G	Leaves	Zhou (2018)
195	Mollchinane	Mollchinanone A	Leaves	Zhou (2018)
196	Mollchinane	Mollchinanone B	Leaves	Zhou (2018)
197	Mollane	Mollanol A	Fruits	Li et al. (2014)
198	Mollane	Mollanol B	Leaves	Zhou (2018)
199	Mollane	Mollanol C	Leaves	Zhou (2018)
200	Mollane	Mollanol D	Leaves	Zhou (2018)
201	Mollane	Mollanol E	Leaves	Zhou (2018)
202	Mollane	Mollanol F	Leaves	Zhou (2018)
203	Mollane	Mollanol G	Leaves	Zhou (2018)
204	Mollane	Mollanol H	Leaves	Zhou (2018)
205	Rhodochinane	Rhodochinanol A	Leaves	Zhou (2018)
206	Rhodochinane	Rhodochinanol C	Leaves	Zhou (2018)
207	Rhodochinane	Rhodochinanol B	Leaves	Zhou (2018)
208	Rhodochinane	Rhodochinanol D	Leaves	Zhou (2018)
209	Rhodochinane	Rhodochinanol E	Leaves	Zhou (2018)
210	Rhodochinane	Rhodochinanol F	Leaves	Zhou (2018)
211	Rhodochinane	Rhodochinanol G	Leaves	Zhou (2018)
212	Rhodochinane	Rhodochinanol H	Leaves	Zhou (2018)
213	2,3-secograyanane	Secomollfoliagein A	Leaves	Zhou (2018)
214	2,3-secograyanane	Secomollfoliagein B	Leaves	Zhou (2018)
215	2,3-secograyanane	Secomollfoliagein C	Leaves	Zhou (2018)
216	2,3-secograyanane	Mollactolide B	Leaves	Zhou (2018)
217	2,3-secograyanane	Mollactolide A	Leaves	Zhou (2018)
218	2,3-secograyanane	Mollactolide C	Leaves	Zhou (2018)
219	2,3-secograyanane	Mollactone D	Leaves	Zhou (2018)
220	2,3-secograyanane	Mollactone E	Leaves	Zhou (2018)
221	Leucothane	Rhodomollein LII	Flowers	Li et al. (2020)
222	Leucothane	Rhodomollein LIII	Flowers	Li et al. (2020)
223	Rhodmollane	Rhodomollein LXIII	Flowers	Song et al. (2025)
224	1,10:2,3-disecograyanane	Mollolide A	Roots	Li et al. (2013)
225	Mollebenzylane	Mollebenzylanol A	Leaves	Zhou et al., 2018a; Zhou (2018)
226	Mollebenzylane	Mollebenzylanol B	Leaves	Zhou et al., 2018a; Zhou (2018)
227	Rhodomollane^a^	Rhodomollin A	Leaves	Li et al. (2016)
228	Rhodomollane^a^	Rhodomollin B	Leaves	Li et al. (2016)
229	Rhodomollane^b^	Rhodomollanol A	Flowers, Leaves	Zheng et al. (2024b), Zhou, et al., 2017b; Zhou (2018)
230	Rhomollane	Rhomollone A	Flowers	Li et al. (2020)
231	Rhodosinoane	Rhodosinoanol A	Leaves	Zhou (2018)
232	Rhodomollsinane	Rhodomollsinone A	Leaves	Zhou (2018)
233	Rhodomollsinane	Rhodobenzylone A	Leaves	Zhou (2018)
234	Mollsinoane	Mollsinoanol A	Leaves	Zhou (2018)

### 5.1 Diterpenoids

Grayanoids are natural diterpenoids that include grayanane-type compounds and their derivatives, most of which are unique to the Ericaceae family. As secondary metabolites, grayanoids are commonly found in the leaves, flowers, and roots of Ericaceae plants, as well as in “mad honey” and are widely known to have medicinal properties. In this review, a total of 17 carbon skeletons from *R. molle* were summarized, including grayanane, 3,4-secograyanane, 5,6-secograyanane, 2,3-secograyanane, kalmane, 1,5-secokalmane, leucothane, 1,10:2,3-disecograyanane, mollane, dimeric grayanoid, rhodomollane, mollebenzylane, 2,3:5,6-disecograyanane, rhodomollsinane, mollchinanane, rhomollane, mollsinoane, and rhodosinoane. Proposed biogenetic pathways are provided in Figure 2.

Grayanane-type diterpenoids predominated, characterized by a distinctive 5/7/6/5 tetracyclic ring system derived from the rearrangement of ent-kaurene. Structurally, these metabolites typically contained oxygenated substituents (e.g., hydroxyl, methoxy, acetoxy, carbonyl, and glycosyl groups) at positions C-2, C-3, C-5, C-6, C-10, C-14, and C-16. Chlorine atoms and epoxide groups were also frequently observed. Structural diversity arose from variations in the type, number, and position of these substituents, as well as the linkage modes of the A/B/C rings. To date, 234 diterpenoids have been isolated from the roots, flowers, fruits, and leaves of *R. molle* (Table 3; Figure 3), comprising 162 grayanane-type diterpenoids (**1**–**162**) and 72 diterpenoids of other structural types (**163**–**234**). Pharmacological studies revealed their analgesic, anti-inflammatory, immunomodulatory, insecticidal, and anti-tumor activities.

### 5.2 Triterpenoids

A total of 59 triterpenoids have been isolated from *R. molle* (Table 4; Figure 4). These metabolites, which are predominantly pentacyclic or tetracyclic in structure, exhibit considerable structural diversity and can be categorized into oleanane **(235–258)**, ursane **(259–285)**, and lupane **(286–293)** types.

**TABLE 4:** Triterpenoids isolated and identified from *Rhododendron molle*.

No.	Classifications	Name	Plant part	References
235	Oleananes	Oleanic acid	Flowers	Zhang (2020)
236	Oleananes	Sumaresinolic acid	Roots	Zhang (2020)
237	Oleananes	Maslinic acid	Roots, flowers	Zhang (2020)
238	Oleananes	2α,3β,24-trihydroxy urs-12-en-28-oic acid	Flowers, roots	Zhang, 2020; Zhang Z. R. (2012)
239	Oleananes	2α,3α,23-trihydroxyolean-12-en-oic acid	Roots	Zhang (2020)
240	Oleananes	Spathodic acid	Roots	Zhang (2020)
241	Oleananes	Marinoid G	Roots	Zhang (2020)
242	Oleananes	Ilexosapogenin A	Roots	Zhang (2020)
243	Oleananes	2α,3α,24-trihydroxyolean-12-en-28-oic acid	Roots	Zhang (2020)
244	Oleananes	Arjunolic acid	Roots	Zhang (2020)
245	Oleananes	2α,3β,24-trihydroxyolean-12-en-oic acid	Roots	Zhang (2020)
246	Oleananes	Taraxerol	Roots	Zhang (2020)
247	Oleananes	2α,3β,23,24-tetrahydroxyolean-12-en-28-oic acid	Roots	Zhang (2020)
248	Oleananes	3,23,24-trinor-2,4-secoolean-12-en-2,4,28-trioic acid	Roots	Zhang (2020)
249	Oleananes	23-nor-2,3-secoolean-4,12-dien-2,3,28-trioic acid	Roots	Zhang (2020)
250	Oleananes	2-methylester-2,3-secolean-4,12-dien-3,28-dioic acid	Roots	Zhang (2020)
251	Oleananes	24β-hydroxy-23α,28-dicarbonyl-2,3-secoolean-12-en-2-oic acid	Roots	Zhang (2020)
252	Oleananes	24-nor-23,28-dicarbonyl-2,3-secoolean-12-en-2-oic acid	Roots	Zhang (2020)
253	Oleananes	2α,3β,24-trihydroxyolean-11α,12α-epoxy-13,28-olide	Roots	Zhang (2020)
254	Oleananes	3β-Hydroxy-D: B-friedo-olean-5-en-3β-ol	Roots	Zhang (2020)
255	Oleananes	Hederagenin	Roots	Zhang (2020)
256	Oleananes	Belleric acid	Roots	Zhang (2020)
257	Oleananes	2α,3β,23,23-Trihydroxy-11α,l2α-epoxyolean-28,13β-olide	Roots	Zhang (2020)
258	Oleananes	Methyl 3β-O-p-methoxy-cinnamoyloxy-2α,23-dihydroxyolean-12-en-28-oate	Roots	Zhang (2020)
259	Ursanes	Ursolic acid	Roots	Zhang (2020)
260	Ursanes	2α,3α,23-trihydroxyurs-12-en-28-oic acid	Roots	Zhang (2020)
261	Ursanes	Pomolic acid	Roots	Zhang (2020)
262	Ursanes	Euscaphic acid	Roots	Zhang (2020)
263	Ursanes	Barbinervic acid	Roots	Zhang (2020)
263	Ursanes	2α,3α,19α,23-tetrahydroxyurs-12-en-28-oic acid	Roots	Zhang (2020)
265	Ursanes	Rotundic acid	Roots	Zhang (2020)
266	Ursanes	19α,23-dihydroxy-3-oxo-urs-12-en-28-oic acid.	Roots	Zhang (2020)
267	Ursanes	Corosolic acid	Roots	Zhang (2020)
268	Ursanes	Asiatic acid	Roots	Zhang (2020)
269	Ursanes	(4R)-23-nor-(2S)-hydroxyl-cyclopenta [4,2-b] furan-3-one-urs-12,20-dien-2-oic acid	Roots	Zhang (2020)
270	Ursanes	23-nor-2,3-secours-4,12-dien-2,3,28-trioic acid	Roots	Zhang (2020)
271	Ursanes	3,23,24-trinor-2,4-secours-12,20-dien-2,4,28-trioic acid	Roots	Zhang (2020)
272	Ursanes	24β-hydroxy-23α,28-dicarbonyl-2,3-secours-12-en-2-oic acid	Roots	Zhang (2020)
273	Ursanes	23-nor-2,3-secours-4,12,20-trien-2,3,28-trioic acid	Roots	Zhang (2020)
274	Ursanes	2-methyl ester-2,3-secours-4,12,20-trien-3,28-dioic acid	Roots	Zhang (2020)
275	Ursanes	2α,3β,23,24-tetrahydroxyurs-12,20-dien-2-oic acid	Roots	Zhang (2020)
276	Ursanes	2α,3β,24-trihydroxyurs-12-en-23α,28-dioic acid.	Roots	Zhang (2020)
277	Ursanes	24β-hydroxy-23α,28-dicarbonyl-2,3-secours-12,20-dien-2-oic acid	Roots	Zhang (2020)
278	Ursanes	2α,3α,19α-trihydroxy-11-oxo-urs-12-en-28-oic acid	Roots	Zhang (2020)
279	Ursanes	13β,28-epoxy-3β,23-dihydroxyursane-12-one.	Roots	Zhang (2020)
280	Ursanes	Micromeric acid	Roots	Zhang (2020)
281	Ursanes	Actinidic acid	Roots	Zhang (2020)
282	Ursanes	2α,3α,23,24-tetrahydroxyurs-12,20-dien-28-oic acid	Roots	Zhang (2020)
283	Ursanes	3β-hydroxyours-12,19(29)-dien-28-oic acid	Roots	Zhang (2020)
284	Ursanes	2α,3α,19α,23-tetrahydroxyours-12,20(30)-dien-28-oic acid	Roots	Zhang (2020)
285	Ursanes	24,30-dihydroxy-urs-3-oxo-12,19-diene-28-oic acid	Roots	Zhang (2020)
286	Lupanes	3α-acetoxy-27-hydroxylup-20(29)-en-28-oicacid methyl ester	Roots	Zhang (2020)
287	Lupanes	Betulinic acid	Roots	Wu et al., 2024; Zhang (2020)
288	Lupanes	Alphitolic acid	Roots	Zhang (2020)
289	Lupanes	Lupenone	Roots	Zhang (2020)
290	Lupanes	Betulin	Roots	Zhang (2020)
291	Lupanes	3-epibetulinic acid	Roots	Zhang (2020)
292	Lupanes	Eucalyptolic acid	Roots	Zhang (2020)
293	Lupanes	3β-O-cis-ferulyl-2a-hydroxy-lup-20(29)-ene-28-oic acid	Roots	Zhang (2020)

### 5.3 Flavonoids

*R. molle* is rich in flavonoids. To date, 26 flavonoids (**294**–**318**) have been reported in *R. molle* (Table 5; Figure 5), including flavonols (**294**–**304**), dihydrochalcones (**305**–**310)**, flavan-3-ols (**311**–**313**), dihydroflavonols (**314**–**315**), flavanones (**316**), and biflavonoids (**317**–**318**).

**TABLE 5:** Flavonoids isolated and identified from *Rhododendron molle*.

No.	Classifications	Name	Plant part	References
294	Flavonols	Quercetin	Flowers	Guo et al. (2024)
295	Flavonols	Quercitrin	Flowers	Guo et al. (2024)
296	Flavonols	Quercetin-3-O-α-L-arabinoside	Flowers	Guo et al. (2024)
297	Flavonols	Quercetin-3-O-β-D-galactoside (Hyperoside)	Flowers	Guo et al. (2024), Song et al. (2022)
298	Flavonols	Quercetin-3-rhamnoside 2^″^-gallate	Flowers	Guo et al. (2024)
299	Flavonols	Kaempferol	Flowers	Guo et al. (2024)
300	Flavonols	Kaempferol-7-O-α-L-rhamnoside	Flowers	Guo et al. (2024)
301	Flavonols	Caryatin	Flowers	Guo et al. (2024)
302	Flavonols	Isorhamnetin	Flowers	Guo et al. (2024)
303	Flavonols	Quercetin-3′-O-glycoside	Fruits	Guo et al. (2024)
304	Flavonols	Myricetin	Fruits	Guo et al. (2024)
305	Dihydrochalcones	Phloretin	Flowers	Cai et al. (2018)
306	Dihydrochalcones	Phlorezin 4′-methyl ether	Flowers	Cai et al. (2018)
307	Dihydrochalcones	Phloretin 4′-yl-β-D-glucopyranoside	Flowers	Cai et al. (2018)
308	Dihydrochalcones	Phloretin 4′-methyl ether	Flowers	Cai et al. (2018)
309	Dihydrochalcones	Phloretin 6′-methyl ether	Flowers	Cai et al. (2018)
310	Dihydrochalcones	Phlorizin	Fruits	Song et al. (2022)
311	Flavan-3-ols	Gallocatechin	Fruits	Song et al. (2022)
312	Flavan-3-ols	(−)-Epicatechin	Fruits	Long (2011)
313	Flavan-3-ols	(+)-Catechin	Roots, fruits	Long (2011)
314	DihydroFlavonols	Taxifolin-3′-O-glucopyranoside	Fruits	Guo et al. (2024)
315	Dihydroflavonols	Dihydromyricetin	Fruits	Guo et al. (2024)
316	Flavones	Vitexin	Flowers	Cai et al. (2018), Guo et al. (2024)
317	Biflavonoids	Proanthocyanidin A2	Fruits	Cai et al. (2018), Guo et al. (2024)
318	Biflavonoids	Dehydroicatechin A	Fruits	Cai et al. (2018)

### 5.4 Lignans

A total of 8 lignans (**319**–**326**) have been reported in *R. molle* (Table 6; Figure 6).

**TABLE 6:** Lignans isolated and identified from *Rhododendron molle*.

No.	Name	Plant part	References
319	7S,8S-threo-4,9,9′-trihydroxy-3,3′-dimethoxy-8-O-4′-neolignan-7-O-β-glucopyranoside	Roots	Zhi et al. (2013)
320	7S,8R-erythro-4,9,9′-trihydroxy-3,3′-dimethoxy-8-O-4′-neolignan-7-O-β-glucopyranoside	Roots	Zhi et al. (2013)
321	7R,8R-threo-4,7,9,9′-tetrahydroxy-3-methoxy-8-O-4′-neolignan-3′-O-β-glucopyranoside	Roots	Zhi et al. (2013)
322	(+)-Lyoniresinol-3α-O-β-glucopyranoside	Roots	Zhi et al. (2013)
323	(−)-Lyoniresinol-3α-O-β-glucopyranoside	Roots	Zhi et al. (2013)
324	(+)-Lyoniresinol	Roots	Zhi et al. (2013)
325	Lyoniresinol-3α-O-β-rhamnopyranoside	Roots, fruits	Zhi et al. (2013)
326	Pinoresinol	Flowers	Zhang Z. R. (2012)

### 5.5 Others

**FIGURE 7:**
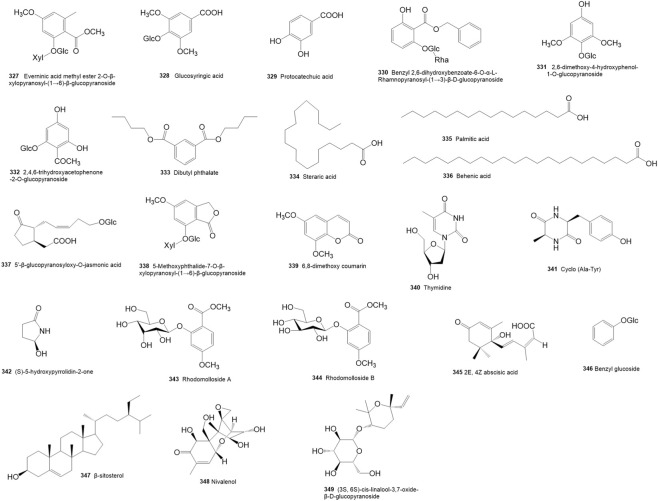
Structures of the other metabolites isolated and identified from *Rhododendron molle*.

Additional metabolites isolated from *R. molle* include phenolic acids (**327**–**332**), fatty acids (**333**–**337**), phenylpropanoids (**338**–**339**), alkaloids (**340**–**342**), and other miscellaneous metabolites (Figure 7; Table 7). However, these metabolites are less reported than the former four types. Among these, it is worth noting that dibutyl phthalate (**333**) is a widely used industrial plasticizer rather than a natural metabolite of plant; it may be detected in plant tissues through absorption or metabolic processes as a result of soil or environmental contamination.

**TABLE 7:** The other metabolites isolated and identified from *Rhododendron molle*.

No.	Classifications	Name	Plant part	References
327	Phenolic acids	Everninic acid methyl ester 2-O-β-xylopyranosyl-(1→6)-β-glucopyranoside	Roots	Bao et al. (2003)
328	Phenolic acids	Glucosyringic acid	Roots	Bao et al. (2003)
329	Phenolic acids	Protocatechuic acid	Roots	Bao et al. (2003)
330	Phenolic acids	benzyl-2,6-dihydroxyl-benzoate-6-O-α-lrhamnopyranosyl-(1→3)-β-D-glucopyranoside	Flowers	Chen et al. (2013)
331	Phenolic acids	2,6-dimethoxy-4-hydroxyphenol-1-O-glucopyranoside	fruits	Cai et al. (2018)
332	Phenolic acids	2,4,6-trihydroxyacetophenone-2-O-glucopyranoside	Fruits	Cai et al. (2018)
333	Fatty acids	Dibutyl phthalate	Flowers	Wang and Fu (2014)
334	Fatty acids	Stearic acid	Flowers, roots	Wu et al. (2024)
335	Fatty acids	Palmitic acid	Roots	Wu et al. (2024)
336	Fatty acids	Behenic acid	Roots	Wu et al. (2024)
337	Fatty acids	5′-β-glucopyranosyloxy-O-jasmonic acid	fruits	Cai et al. (2018)
338	Phenylpropanoids	5-Methoxyphthalide-7-O-β-xylopyranosyl-(1→6)-β-glucopyranoside	Roots	Bao et al. (2003)
339	phenylpropanoids	6,8-dimethoxy coumarin	Flowers	Zhang (2012)
340	Alkaloid	Thymidine	Flowers	Guo et al. (2025)
341	Alkaloid	Cyclo (Ala-Tyr)	Flowers	Guo et al. (2025)
342	alkaloid	(S)-5-hydroxypyrrolidin-2-one	Flowers	Guo et al. (2025)
343	Glycosides of methyl everninate	Rhodomolloside A	Flowers	Yang et al. (2022)
344	Glycosides of methyl everninate	Rhodomolloside B	Flowers	Yang et al. (2022)
345	Sesquiterpenes	2E,4Z-abscisic acid	Flowers	Wang and Fu (2014)
346	Simple Glycosides	Benzyl glucoside	Flowers	Wang and Fu (2014)
347	Phytosterols	β-sitosterol	Roots, Flowers	Wang and Fu (2014)
348	Terpenoids	Nivalenol	Flowers	Guo et al. (2025)
349	Monoterpenes	(3S, 6S)-cis-linalool-3,7-oxide-β-D-glucopyranoside	Flowers	Guo et al. (2025)

## 6 Pharmacological activities

**FIGURE 8:**
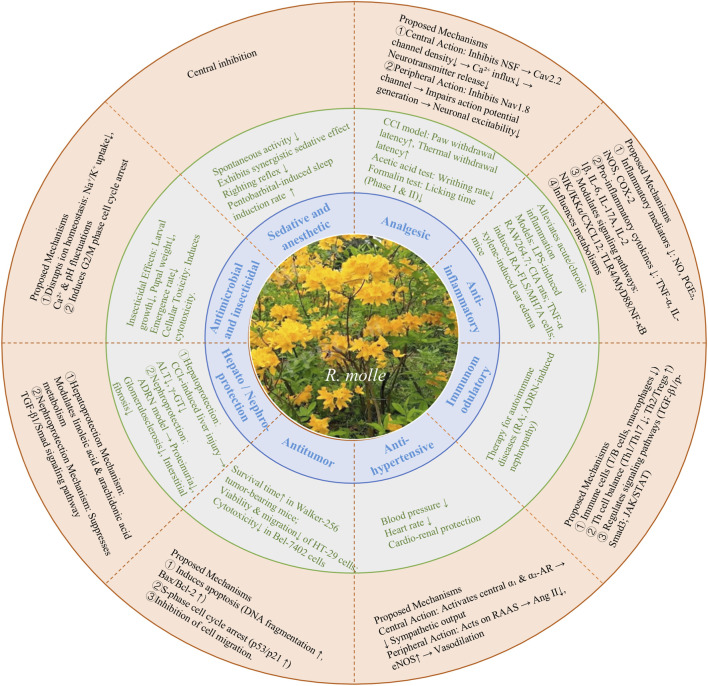
The pharmacological activities of *Rhododendron molle*.

In recent years, numerous pharmacological investigations of *R. molle* have been conducted, revealing that its extracts and isolated metabolites possess diverse pharmacological effects, including analgesic, anti-inflammatory, immunomodulatory, antihypertensive, antitumor, sedative and anesthetic, antimicrobial and insecticidal, as well as hepato-protective and reno-protective activities. These pharmacological activities are summarized in Figure 8. Among these pharmacological effects, analgesic activity for grayanane-type diterpenoids attracts considerable attention of pharmacologists, and some metabolites such as GTX-I (**117**) and GTX-III (**10**) exhibit more significant or equivalent analgesic activities to that of morphine.

### 6.1 Analgesic activities

*R. molle* has long been recognized as a valuable botanical source of analgesic metabolites. The analgesic effects of extracts and metabolites isolated from *R. molle* are summarized in Table 8. The acetic acid-induced writhing test, a well-established, sensitive, and predictive animal model for acute pain, was widely used to evaluate analgesic activity. The diterpenoid fraction of *R. molle* roots have been shown to effectively reduce acetic acid-induced writhing episodes in mice (Yao, 2019), indicating that diterpenoids may be the major analgesic metabolites of *R. molle*. A variety of diterpenoids isolated from *R. molle* have demonstrated significant analgesic activity in the acetic acid-induced writhing test. Among all the tested metabolites, RM-X (**113**), RJ-III (**16**), RJ-VI (**118**), rhodomollinol A (**180**) and rhodomollinol B (**115**), RM-LXVIII (**151**), RM-XIV (**178**), 2-O-methylrhodojaponin VI (**1**), kalmanol (**177**), GTX-I (**117**) and GTX-III (**10**) showed the most remarkable analgesic activity (Li et al., 2013; Li et al., 2020). Notably, at a dose of 0.6 mg/kg, RJ-VI (**118**) could reduce writhe frequency by 63.58%, which was 157.38% of the efficacy of 300 mg/kg aspirin (Wang et al., 2024). Kalmanol (**177**) achieved an inhibition rate of 34.3% at 0.4 mg/kg (Li et al., 2013; Li et al., 2020), while RM-LXVIII (**151**) and RJ-VI (**118**) yielded inhibition rates of 66.3% and 82.9%, respectively, at 0.2 mg/kg (Wang et al., 2010; Wang et al., 2025; Zhu et al., 2024). RJ-III (**16**) exhibited significant analgesic activity even at lower doses, inhibiting 73.7% of writhing episodes at 0.08 mg/kg (Yang et al., 2022a). Most notably, multiple diterpenoids maintained potent analgesic effects at an ultra-low dose of 0.04 mg/kg: RM-X (**113**), RJ-VI (**118**), and RJ-VII (**5**) produced inhibition rates ranging from 61.7% to 85.8% (Zheng et al., 2024a); GTX-I (**117**) and GTX-III (**10**) produced substantial inhibition with rates of 71.5% and 69.3%, respectively (Sun et al., 2019); and rhodomollinols A (**180**) and B (**115**), along with RM-X (**113**) and 2-O-methylrhodojaponin VI (**1**), exhibited inhibition rates of 51.8%, 48.0%, 61.7%, and 60.0%, respectively (Zheng et al., 2024b).

**TABLE 8:** The analgesic effects of the extracts and metabolites isolated from *Rhododendron molle*.

Phytochemicals	Model	Positive control	Dose/Duration	Results	References
Diterpenoid fraction of *R. molle* roots	Acetic acid-induced writhing test	Indomethacin enteric-coated tablets	0.13, 0.4, and 1.2 mg/kg (p.o.)	Inhibited the writhes by 33.5%, 53.3%, and 55.3%, respectively	Yao (2019)
Diterpenoid fraction of *R. molle* roots	Hot-plate test	Morphine hydrochloride injection	1.20 mg/kg (i.p.), for 30 min	Elevated the pain threshold in mice	Yao (2019)
Metabolites **79**, **34**, **222**, **120**, **181**	Acetic acid-induced writhing test	—	20 mg/kg (i.p.), for 30 min	Inhibited the writhes by 41.9%–91.6%	Li et al. (2020)
Metabolites **1**, **37**	Acetic acid-induced writhing test	—	2 mg/kg (i.p.)	Inhibited the writhes by 46.0% and 39.4%, respectively	Li et al. (2020)
Metabolites **154**, **152, 158**, **222**, **174**, **180**, **115**, **145**, **148**, **1**	Acetic acid-induced writhing test	Morphine	1.0 mg/kg (i.p.), for 30 min	Inhibited the writhes exceeded 50%	Song et al. (2025), Zheng et al. (2024b)
Metabolites **180**, **115**, **113**, **148**	Acetic acid-induced writhing test	Morphine	0.04 mg/kg (i.p.), for 30 min	Inhibited the writhes by 51.8%, 48.0%, 61.7%, and 60.0%, respectively	Zheng et al. (2024b)
Metabolites **151**, **120, 108**, **25**	Acetic acid-induced writhing test	—	1.0 mg/kg (i.p.), for 30 min	Inhibited the writhes by 66.8%–91.7%	Wang et al. (2025)
Metabolites **108**, **25**	Acetic acid-induced writhing test	—	5.0 mg/kg (i.p.), for 30 min	Inhibited the writhes by 68.4%–83.4%	Wang et al. (2025)
Secorhodomollolide D (**187**)	Acetic acid-induced writhing test	—	5 mg/kg (i.p.), for 30 min	Inhibited the writhes by 35%	Wang et al. (2010)
Mollolide A (**224**)	Acetic acid-induced writhing test	—	5 mg/kg (i.p.), for 30 min	Inhibited the writhes by 73%	Li et al. (2013)
RJ-III (**16**)	Acetic acid-induced writhing test	Morphine	0.08 mg/kg (i.p.), for 30 min	Inhibited the writhes by 73.7%	Li et al. (2015)
	AceticAcetic acid-induced writhing test	Morphine	0.02, 0.04, 0.08 mg/kg (i.p.), for 30 min	ID_50_ = 46.9 mg/kg (34.6–67.6, 95% confidence)	Li et al. (2015)
	Formalin test	Morphine	0.02, 0.04, 0.08 mg/kg (i.p.), 0–5 min for phase I and 15–30 min for phase II	Caused a significant reduction in the time spent licking during both phases I and II.	Li et al. (2015)
	STZ-induced diabetic neuropathy	Gabapentin	0.8 mg/kg (p.o.)	Reversed mechanical hyperalgesia, with potency ∼100 times greater than gabapentin	Li et al. (2015)
	hot plate test	Aspirin	0.20 mg/kg (p.o.), for 45 min	Extended the latency time, reaching a maximum analgesic activity of 82.4% at 0.20 mg/kg	Yang et al. (2022a)
​	tail-immersion test	Aspirin	0.20 mg/kg (p.o.), for 10 s	Extended tail-flick latency, achieving 60% of the maximum possible analgesic effect at 0.20 mg/kg	Yang et al. (2022a)
	Acetic acid-induced writhing test	Aspirin	0.20 mg/kg (p.o.), for 30 min	Inhibited the writhes by 67.9%	Yang et al. (2022a)
	chronic constriction injury model (CCI)	Gabapentin	0.3 mg/kg (p.o.), for 60 min	Increased the paw withdrawal latency	Yang et al. (2022a)
	Acetic acid-induced writhing test	Morphine	0.2 mg/kg (i.p.), for 30min	Inhibited the writhes by 97.7%	Zheng et al. (2024a)
RJ-VI (**118**)	Acetic acid-induced writhing test	Morphine	0.02, 0.04, 0.08 mg/kg (i.p.), for 30 min	Inhibited the writhes by 64.2% ID_50_ = 72.7 μg/kg (350.2–132.4, 95% confidence)	Li et al. (2015)
	formalin test	Morphine	0.02, 0.04 and 0.08 mg/kg (i.p.), 0–5 min for phase I and 15–30 min for phase II	Caused a significant reduction in the time spent licking during both phases I and II	Li et al. (2015)
	STZ-induced diabetic neuropathy	Gabapentin	0.2, 0.4, 0.8 mg/kg (p.o.)	Reversed mechanical hyperalgesia, with potency ∼100 times greater than gabapentin	Li et al. (2015)
	CCI model	—	i.p.	Alleviated neuropathic pain by inhibiting NSF-mediated membrane trafficking of the N-type voltage-gated calcium channel Cav2.2, thereby reducing the membrane abundance and current intensity of Cav2.2	Chen et al. (2023)
	Acetic acid-induced writhing test	Aspirin	0.3, 0.6 mg/kg (p.o.), for 30 min	inhibited the writhes by 36.09% and 63.58%, respectively	Wang et al. (2024)
	formalin test	Aspirin	0.3, 0.6 mg/kg (p.o.), within 0–5 min (Phase I) and 15–35 min (Phase II)	Prolonged the response latency in both Phase I and II	Wang et al. (2024)
	Acetic acid-induced writhing test	Morphine	0.2, 0.04 mg/kg (i.p.), for 30 min	Inhibited the writhes by 60.0% and 85.8%, respectively.	Zheng et al. (2024a)
	Acetic acid-induced writhing test	—	0.2 mg/kg (i.p.), for 30 min	Inhibited the writhes by 82.9%	Wang et al. (2025)
Kalmanol (**177**)	Acetic acid-induced writhing test	—	0.4 mg/kg (i.p.), for 30 min	Inhibited the writhes by 34.3%	Li et al. (2020)
Molleblossomin F (**132**)	Acetic acid-induced writhing test	Morphine	0.2 mg/kg (i.p.), for 30 min	Inhibited the writhes by 51.4%	Zheng et al. (2024a)
Molleblossomin C (**129**)	Acetic acid-induced writhing test	Morphine	0.2 mg/kg (i.p.), for 30 min	Inhibited the writhes by 68.2%	Zheng et al. (2024a)
RM-X (**113**)	Acetic acid-induced writhing test	Morphine	0.2, 0.04 mg/kg (i.p.), for 30 min	Inhibited the writhes by 94.1% and 61.7%, respectively.	Zheng et al. (2024a)
RJ-XXVI (**85**)	Acetic acid-induced writhing test	Morphine	0.2 mg/kg (i.p.), for 30 min	Inhibited the writhes by 66.9%	Zheng et al. (2024a)
RJ-VII (**5**)	Acetic acid-induced writhing test	Morphine	0.04 mg/kg (i.p.), for 30 min	Inhibited the writhes by 64.6%	Zheng et al. (2024a)
RM- LVII (**153**)	Acetic acid-induced writhing test	Morphine	0.2 mg/kg (i.p.), for 30 min	Inhibited the writhes by 77.2%	Song et al. (2025)
RM-LIX (**155**)	Acetic acid-induced writhing test	Morphine	0.2 mg/kg (i.p.), for 30 min	Inhibited the writhes by 71.5%	Song et al. (2025)
RM-LXVIII (**151**)	Acetic acid-induced writhing test	—	0.2 mg/kg (i.p.), for 30 min	Inhibited the writhes by 66.3%	Wang et al. (2025)
RM-XIV (**178**)	Acetic acid-induced writhing test	Morphine	6, 20 mg/kg (i.p.), for 30 min	Inhibited the writhes by 72.9% and 100%, respectively	Zhu et al. (2024)
GTX-III (**10**)	Acetic acid-induced writhing test	Morphine	0.08 mg/kg (i.p.), for 30 min	Inhibited the writhes by 55.6%	Li et al. (2015)

p.o., oral administration; i.p., intraperitoneal injection.

The formalin test is a method used to assess acute and persistent inflammatory pain, in which the phase I response corresponds to acute neurogenic pain, whereas the phase II response involves an inflammatory process. RJ-III (**16**) and RJ-VI (**118**) each significantly reduced the time spent licking during both phases I and II, indicating their efficacy against both acute and persistent pain (Li et al., 2015). Specifically, RJ-III exhibited a maximum analgesic activity of 66.9% at 0.10 mg/kg in phase II (Yang et al., 2022a); at doses of 0.3 and 0.6 mg/kg, it also prolonged the response latency in both phases I and II (Wang et al., 2024). The hot plate test and tail-immersion test are commonly used to evaluate analgesic activity against thermal nociception. In the hot plate test, RJ-III (**16**) significantly extended the latency time at a dose of 0.2 mg/kg, reaching a maximum analgesic activity of 82.4% (Yang et al., 2022a). Similarly, in the tail-immersion test, RJ-III (**16**) significantly prolonged tail-flick latency within 75 min post-administration at 0.2 mg/kg, achieving 60% of the maximum possible analgesic effect (Yang et al., 2022a). The streptozotocin (STZ)-induced diabetic mouse model, a well-established model of diabetic peripheral neuropathic pain (DPNP), was employed to evaluate neuropathic pain. When administered orally, RJ-III (**16**) and RJ-VI (**118**) produced dose-dependent reversal of mechanical hyperalgesia in diabetic mice, with potencies approximately 100 times that of gabapentin (Li et al., 2015). This finding highlights the potential of *R. molle*-derived diterpenoids as novel therapeutic agents for neuropathic pain. CCI is a composite model of mainly neuralgia accompanied by inflammatory pain, both of which can simulate clinical peripheral nerve injury. In CCI-induced pain experiments, 0.3 mg/kg RJ-III (**16**) significantly increased the paw withdrawal threshold latency (PTWL) and achieved the maximum inhibition rate of hyperalgesia at 45–60 min (Yang et al., 2022a). RJ-VI (**118**) also effectively reduced mechanical pain in the CCI test, further confirming its efficacy against neuropathic pain (Wang et al., 2024).

Mechanistic studies revealed that RJ-VI (**118**) alleviates neuropathic pain by inhibiting N-ethylmaleimide-sensitive fusion protein (NSF)-mediated membrane trafficking of the N-type voltage-gated calcium channel Cav2.2, thereby reducing the membrane abundance and current intensity of Cav2.2 (Chen et al., 2023). The voltage-gated sodium channel Nav1.8, a tetrodotoxin-resistant channel, is predominantly expressed in nociceptive neurons of the dorsal root ganglia and trigeminal ganglia. Studies demonstrated that RJ-VI (**118**) showed inhibitory effect on Nav1.8, and the I/Io% of the 100 μM was 72.20%, suggesting RJ-VI (**118**) was closely associated with its direct inhibitory action on Nav1.8 channel function (Wang et al., 2024). These results indicate that extracts of *R. molle* and numerous diterpenoids derived from it exhibit significant analgesic activity. However, inconsistencies in extract types, dosage regimens, and positive controls across studies impede direct comparisons of the activities of different compounds and complicate the establishment of structure–activity relationships. Furthermore, current research on analgesic mechanisms has focused on RJ-VI (**118**), particularly their involvement with Cav2.2 and Nav1.8 ion channels, while many other diterpenoids with pronounced analgesic activity remain uncharacterized with respect to their mechanisms.

### 6.2 Anti-inflammatory activities

Inflammation is a key pathological process involved in a variety of diseases, including RA, cancer, and chronic pain. Much evidence has demonstrated that the different plant parts of *R. molle* possess anti-inflammatory activities. Regarding the roots, *in vivo* studies have shown that both water extracts and the 95% ethanol fraction inhibit histamine-induced joint swelling and xylene-induced ear edema, and reduce histamine-induced capillary permeability (Yao et al., 2019). *In vitro*, the same preparations inhibited IL-1β production in LPS-stimulated RAW264.7 cells and suppressed inflammatory cell proliferation as well as the levels of IL-1β, IL-17A, IL-6, IL-2, and IFN-γ in TNF-α-induced rheumatoid arthritis fibroblast-like synoviocytes (RA-FLS) through modulation of the EGFR, NF-κB, AKT, and JNK pathways (Yao et al., 2019; Liu M. L. et al., 2023). The leaves of *R. molle* have also been reported to exhibit anti-inflammatory activity. Methanol extracts of the leaves inhibited the production of the pro-inflammatory mediators TNF-α, IL-1β, IL-6, and NO in LPS-stimulated RAW264.7 cells (Zong et al., 2020). Subsequent studies extended these findings by evaluating different solvent fractions (chloroform, ethyl acetate, and n-butanol) of the leaves, demonstrating their efficacy in suppressing hind paw swelling and reducing the arthritis index in CIA rats. These fractions also inhibited the production of TNF-α, IL-1β, IL-6, COX-2, and NO in LPS-induced RAW264.7 cells, with the chloroform fraction exhibiting the most potent activity (Luo et al., 2023a; Luo et al., 2023b). With regard to the flowers, ethanol extracts were found to inhibit xylene-induced ear edema in mice (Guo et al., 2020). A more comprehensive study demonstrated that the powder of *R. molle* flowers reduced the levels of IL-1β, IL-6, and TNF-α and alleviated bone destruction in CIA rats, while four targets (AKR1B1, TPH1, CYP1A1, and CYP1A2) were identified, along with metabolites (D-glucose, D-mannose, L-tryptophan, 11-deoxycorticosterone, and 17α-hydroxyprogesterone) involved in steroid hormone biosynthesis, tryptophan metabolism, and galactose metabolism (Guo et al., 2024). For the fruits, the diterpenoid fraction reduced paw swelling and arthritis index, and inhibited inflammatory cell infiltration and serum levels of TNF-α, IL-1β, and IL-6 in CIA rats (Yao, 2019).

Diterpenoids are recognized as the primary active metabolites responsible for the anti-inflammatory effects of *R. molle*. At a concentration of 20 μM, rhodomollosides A (**343**) and B (**344**) inhibited NO production in LPS-induced RAW264.7 cells by 75.8% and 86.4%, respectively (Yang et al., 2022). A study identified 11 diterpenoids that significantly suppressed NO production in LPS-induced RAW264.7 cells, with IC_50_ values ranging from 2.8 to 35.4 μM, including mollfoliagein C (**50**), RJ-I (**14**), RJ-II (**17**), RJ-III (**16**), RJ-VI (**118**), and RJ-VII (**5**), rhodomolin I (**46**), GTX-XVIII (**98**), RM-XI (**120**), 2-O-methylrhodomollein XI (**3**), and 6-O-acetylrhodomollein XXI (**7**). Among them, RJ-II (**17**) exhibited the most potent activity (Zhou et al., 2018b). RJ-II (**17**) ameliorated inflammatory symptoms in CIA mice, and reduced levels of NO, PGE_2_, IL-1β, IL-6, and MMP-1 in TNF-α-induced RA-FLSs and blocked activation of the Akt, NF-κB, and TLR4/MyD88 signaling pathways in MH7A cells (Kong et al., 2020). RJ-III (**16**) inhibited xylene-induced ear edema in mice and reduced IL-1β levels in LPS-induced RAW264.7 cells (Yao et al., 2019). In CIA rats, RJ-III (**16**) decreased the arthritis index, suppressed synovial hyperplasia and inflammatory cell infiltration, and reduced serum levels of inflammatory cytokines (TNF-α, IL-1β, IL-6, IL-17) and matrix metalloproteinases (MMP-2, MMP-9) by inhibiting the TLR4/MyD88/NF-κB pathway. *In vitro*, RJ-III (**16**) restrained abnormal proliferation and inflammatory responses of RA-FLSs *via* modulation of the Wnt1/Dvl1/β-catenin pathway (Liu et al., 2023). Furthermore, RJ-III (**16**) alleviated joint cartilage damage, bone erosion, and angiogenesis in CIA rats, and inhibited the proliferation, migration, invasion, and tube formation of TNF-α-induced human umbilical vein endothelial cells (HUVECs) *in vitro* by regulating the NIK/IKKα/CXCL12 signaling axis (Liu et al., 2023). Recently, RJ-III (**16**) was shown to reduce cartilage tissue damage in a rat model of post-traumatic osteoarthritis, as evidenced by lower Mankin scores, improved levels of BGP, ALP, and CTX-I, and decreased release of IL-1β, IL-6, and TNF-α. This protective effect was associated with regulation of the Bcl-2/Bax/caspase signaling pathway and inhibition of chondrocyte apoptosis (Liu et al., 2024). The anti-inflammatory activities of extracts and metabolites isolated from *R. molle* are summarized in Table 9. The clinical use of *R. molle* in treating RA has been pharmacologically supported by its anti-inflammatory activity, with numerous diterpenoids demonstrating significant anti-inflammatory effects. However, mechanistic investigations have primarily focused on RJ-II (**17**) and RJ-III (**16**), while studies on other diterpenoids with notable anti-inflammatory activity remain largely confined to assessing inhibition of NO or inflammatory cytokine production, lacking systematic mechanistic exploration.

**TABLE 9:** The anti-inflammatory activities of the extracts and metabolites isolated from *Rhododendron molle*.

Phytochemicals	*In vitro* or *in vivo*	Model	Positive control	Results	References
Water extracts of *R. molle* roots	*In vivo*	Histamine-induced arthritis rats	Tripterygium wilfordii tablets	Suppressed joint swelling, subcutaneous inflammation, and inflammatory cell proliferation at the dose of 0.65, 1.3 and 2.6 g/kg	Mei et al. (2023)
	*In vivo*	Histamine-induced vascular permeability rabbits	Tripterygium wilfordii tablets	Reduced capillary permeability at the dose of 1.7 and 2.6 g/kg	Mei et al. (2023)
95% ethanol fraction of *R. molle* roots	*In vitro*	LSP-induced RAW264.7 cells	Tripterygium glycosides	Inhibited IL-1β production (IC50 = 9.81 μg/mL)	Yao et al. (2019)
Water extracts and 95% ethanol fraction of *R. molle* roots	*In vivo*	Xylene-induced ear edema mice	Tripterygium glycosides	Inhibited ear swelling with inhibition rates of 9.21% (water extract, 61.88 mg/kg) and 8.56% (95% ethanol fraction, 8.85 mg/kg)	Yao et al. (2019)
Water extracts of *R. molle* roots	*In vitro*	TNF-α induced RA-FLS cells	Tripterygium glycosides	Inhibited inflammatory cell proliferation and serum IL-1β, IL-17A, IL-6, IL-2, and IFN-γ, through modulation of the EGFR, NF-κB, AKT, and JNK pathways	Liu et al. (2023)
Diterpenoid fraction of *R. molle* fruits	*In vivo*	CIA model rats	Tripterygium glycosides	Reduced paw swelling, arthritis index, and inhibited inflammatory cell infiltration and serum TNF-α, IL-1β, IL-6 at a dose of 0.6 mg/kg/d	Yao (2019)
Methanol extracts of *R. molle* leaves	*In vitro*	LSP-induced RAW264.7 cells	—	Inhibited the pro-inflammatory mediator and cytokines TNF-α, IL-1β, IL-6, and NO	Zong et al. (2020)
Chloroform extract, Ethyl acetate extract , n-butanol extract from *R. molle* leaves	*In vivo*	CIA model rats	Tripterygium glycosides	Inhibit hind paws swelling and reduce arthritis index in arthritis rats	Luo et al. (2023a)
	*In vitro*	LSP-induced RAW264.7 cells	—	Inhibited the levels of TNF-α, IL-1β, IL-6, COX-2 and NO, and chloroform extract showed the best inhibitory activity	Luo et al., 2023a; Luo (2023)
Ethanol extracts of *R. molle* flowers	*In vivo*	Xylene-induced ear edema mice	—	At doses of 1.2 and 0.6 g/kg, the swelling inhibition rates were 73.25% and 58.39%, respectively	
Powders of *R. molle* flowers	*In vivo*	CIA model rats	Tripterygium glycosides	Inhibited the levels of IL-1β, IL-6, and TNF-α and alleviated bone destruction at a dose of 0.8 g/kg, while four targets (AKR1B1, TPH1, CYP1A1, and CYP1A2) were identified, along with metabolites (D-glucose, D-mannose, L-tryptophan, 11-deoxycorticosterone, and 17α-hydroxyprogesterone) involved in steroid hormone biosynthesis, tryptophan metabolism, and galactose metabolism	Guo et al. (2024)
Metabolites **50**, **14**, **46**, **98**, **118**, **220**, **5**, **3**, **7**	*In vitro*	LSP-induced RAW264.7 cells	Dexamethasone	Inhibited NO production (IC50 = 3.5–35.4 μM)	Zhou et al. (2018b)
Metabolites **243**, **344**	*In vitro*	LSP-induced RAW264.7 cells	—	Inhibited NO production by 75.8% and 86.4%, respectively	Yang et al. (2022)
Metabolites **253, 254, 280**	*In vitro*	LSP-induced RAW264.7 cells	—	Inhibited TNF-α production by 32.06%–39.69%	Zhang (2020)
RJ-III (**16**)	*In vitro*	LSP-induced RAW264.7 cells	Dexamethasone	Inhibited NO production (IC50 = 7.0 μM)	Zhou et al. (2018b)
	*In vivo*	Xylene-induced ear edema mice	Tripterygium glycosides	Reduced the degree of ear swelling with an inhibition rate of 29.7% at a dose of 0.24 mg/kg	Yao et al. (2019)
	*In vitro*	LSP-induced RAW264.7 cells	Tripterygium glycosides	Inhibited TNF-α production (IC50 = 1.87 μg/mL), and IL-1β production (IC50 = 1.93 μg/mL)	Yao et al. (2019)
	*In vitro*	TNF-α induced RA-FLS cells	Tripterygium glycosides	Downregulated IL-6, IL-8, CCL2, and CCL5 concentrations, upregulated IL-10 and IL-4 contents, and downregulated the expressions of Wnt1, β-catenin and Dvl1	Liu et al. (2023)
	*In vivo*	CIA model rats	Tripterygium glycosides	Inhibited malignant hyperplasia and inflammatory cell infiltration in the synovial tissue, and reduced the levels of TNF-α, IL-1β, IL-6, IL-17, VEGF, MMP-2, MMP-9, TLR4, MyD88, and the p-NF-κB/NF-κB	Liu et al. (2023)
	*In vivo*	CIA model rats	Tripterygium glycosides	Suppressed cartilage damage and bone erosion, and inhibited expressions of platelet endothelial CD31 and VEGF to decrease vascular density	Liu et al. (2023)
	*In vitro*	TNF-α induced HUVECs cells	—	Suppressed the proliferation, migration, invasion, and angiogenesis of HUVECs, and decreased the levels of IL-6, IL-1β, and TNF-α	Liu M. L. et al. (2023)
RJ-II (**17**)	*In vitro*	LSP-induced RAW264.7 cells	Dexamethasone	Inhibited NO production (IC50 = 2.8 μM)	Zhou et al. (2018b)
	*In vitro*	TNF-α induced MH7A cells	—	Reduced the viability of MH7A cells, suppressed the Akt, NF-κB, and TLR4/MyD88 pathways, downregulated the expression of IL-1β, IL-6, and MMP-1, and decreased the levels of NO and PGE2	Kong et al. (2020)
	*In vivo*	CIA mice	—	Reduced the progression of foot thickening and the arthritis score, and serum levels of IL-1β, IL-6, and MMP-1	Kong et al. (2020)

### 6.3 Immunomodulatory activities

*R. molle* exhibited notable immunosuppressive activities, primarily mediated through inhibition of lymphocyte proliferation and modulation of inflammatory cytokine secretion (Table 10). Early studies in 1995 demonstrated that extracts of *R. molle* roots inhibited T-cell proliferation induced by nitrochlorobenzene and mitigated cutaneous hypersensitivity reactions in mice (Mei et al. 2023). In chronic immune complex glomerulonephritis rats, *R. molle* roots reduced proteinuria, improved renal function, decreased IκB mRNA degradation in renal tissue, and inhibited NF-κB activation. Similarly, the root extract reduced proteinuria, alleviated podocyte injury and glomerulosclerosis, and suppressed interstitial fibrosis in adriamycin (ADRN)-induced nephropathy mice (Qiu et al., 2019). The diterpenoid fraction of *R. molle* fruits significantly attenuated inflammatory symptoms in CIA rats, associated with suppression of aberrant T- and B-lymphocyte proliferation and inhibition of pro-inflammatory cytokines IL-6, IL-1β, and TNF-α (He et al., 2021). Both *in vivo* and *in vitro* studies demonstrated that this diterpenoid fraction regulated Th cell polarization *via* the JAK/STAT pathway and inhibited Th1- and Th17-mediated immune responses, thereby blocking RA pathology (Qin et al., 2022).

**TABLE 10:** The immunomodulatory of the extracts and metabolites isolated from *Rhododendron molle*.

Phytochemicals	*In vitro* or *in vivo*	Model	Positive control	Results	References
Water extract of *R. molle* roots	*In vivo*	DNCB-induced skin hypersensitivity mice	Tripterygium wilfordii tablets	Inhibited the proliferation of T lymphocytes and hypersensitivity reactions	Mei et al. (2023)
	*In vivo*	Chronic immune complex glomerulonephritis rats	—	Reduced proteinuria, improved renal function, decreased IκB mRNA degradation in renal tissue, and inhibited NF-κB activation	Liu et al. (2023)
Ethanol extract of *R. molle* roots	*In vitro*	ConA-stimulated rats	—	Inhibited the proliferation of B and T lymphocytes	Liu et al. (2023)
	*In vivo*	ADRN-induced nephropathy mice	Captopril	Reduced proteinuria, alleviated podocyte injury and glomerulosclerosis, and suppressed interstitial fibrosis at a dose of 60 mg/kg	Qiu et al. (2019)
Diterpenoid fraction of *R. molle* roots	*In vivo*	CIA model rats	Tripterygium glycosides	Alleviated inflammatory symptoms and inhibited the proliferation of the proliferation of B and T lymphocytes	Yao (2019)
Diterpenoid fraction of *R. molle* fruits	*In vivo*	CIA model rats	Tripterygium glycosides	Alleviated inflammatory symptoms, inhibited the proliferation of B and T lymphocytes, decreased the levels of IL-6, IL-1β and TNF-α	He et al. (2021)
	*In vivo*	LPS-induced endotoxin shock mouse	Dexamethasone	Increased the mouse survival rate, decreased the proportion of T helper cells (Th1 and Th17), and increased the proportion of Th2 and regulatory T cells (Tregs), inhibited the secretion of TNF-α, IL-1β, and IL-6, reduced the levels of phosphorylated STAT1 and STAT3 at a dose of 0.2 mg/mL	Qin et al. (2022)
	*In vivo*	Th1 and Th17 cell polarization models	Dexamethasone	Inhibited Th1 and Th17 differentiation from naive CD4^+^ T cells, and suppressed Th1 differentiation by downregulating of STAT1 and STAT4 activity, reducing IFN-γ and T-bet mRNA expression, and inhibited Th17 differentiation by decreasing STAT3 phosphorylation, leading to reduced IL-17a, IL-17f, and RORγt mRNA expression at a dose of at 10 μg/mL	Qin et al. (2022)
RJ-III (**16**)	*In vivo*	CIA rats	Tripterygium glycosides	Alleviated inflammatory symptoms, inhibited the proliferation of B and T lymphocytes, and decreased the levels of IL-1β and TNF-α	Yao et al. (2019)
Metabolites **205**, **207**	*In vitro*	ConA-induced splenic T lymphocyte proliferation	—	Inhibited the proliferation of B lymphocytes	Zhou (2018)
Metabolites **205**, **207**	*In vitro*	LPS-induced splenic B lymphocyte proliferation	—	Inhibited the proliferation of T lymphocytes	Zhou (2018)
RJ-II (**17**)	*In vivo*	ADRN-induced nephropathy mice	—	Reduced NF-ĸB phosphorylation, interstitial infiltrated CD4^+^ T cells, CD8^+^ T cells, and CD68^+^ macrophages, downregulated TGF-β1 and p-Smad3 protein expressions at a dose of 0.04 mg/kg	Qiu et al. (2019)

Some diterpenoids isolated from *R. molle* also exhibited immunoregulatory effects. Rhodochinanols A (**205**) and B (**207**) inhibited proliferation of ConA-induced T cells and LPS-induced B cells from mouse splenocytes (Zhou, 2018). RJ-III (**16**) alleviated paw swelling in CIA rats, suppressed abnormal T- and B-cell proliferation, and reduced serum IL-1β and TNF-α levels (Yao et al., 2019). Pharmacokinetic studies indicated that orally administered RJ-III (**16**) was widely distributed in the thymus and spleen, providing a basis for its direct immunomodulatory effects. RJ-II (**17**) not only improved renal pathology in ADRN-induced nephropathy mice but also reduced infiltration of CD4^+^ and CD8^+^ T cells and CD68^+^ macrophages in the renal interstitium, associated with inhibition of NF-κB p65 phosphorylation and downregulation of the TGF-β1/p-Smad3 pathway (Qiu et al., 2019). Thus, *R. molle* and its diterpenoids exhibit evident immunomodulatory activity; however, mechanistic understanding remains limited. Future research should focus on elucidating their molecular targets and cellular selectivity, which holds promise for the development of novel immunomodulatory agents.

### 6.4 Anti-hypertensive activities

Modern pharmacological studies have demonstrated that *R. molle* and its metabolites exhibit notable anti-hypertensive activities. Early investigations revealed that while intravenous administration of an ethanolic extract of *R. molle* failed to induce hypotensive effect, intracerebroventricular injection of the same dose produced a significant blood pressure reduction, suggesting that its anti-hypertensive mechanism primarily involves the central nervous system (CNS). Further studies showed that this central hypotensive effect was partially antagonized by intracerebroventricular prazosin (an α1-adrenoceptor antagonist) and completely abolished by yohimbine (an α2-adrenoceptor antagonist), indicating that activation of central α-adrenoceptors underlies the mechanism (Cai et al., 2018).

RJ-III (**16**) has been investigated for the management of supraventricular tachyarrhythmias and hypertension due to its bradycardic and hypotensive properties (Chinese Pharmacopoeia Commission, 1977). Pharmacological studies further demonstrated that RJ-III (**16**) exerts antihypertensive, heart rate-lowering, and renoprotective effects in SHR, potentially through reduction of angiotensin II (Ang II) levels and upregulation of endothelial nitric oxide synthase (eNOS) expression. Preliminary studies on GTX-III (**10**) indicated an inverse relationship between dose and changes in blood pressure and heart rate at concentrations of 200, 400, and 800 μg/kg (Türkmen et al., 2013).

### 6.5 Sedative and anesthetic activities

In folk practices, *R. molle* flowers were soaked to prepare liquid extracts used as Insecticides. One study found that 0.2 mg/kg RJ-II (**17**) induced a loss of the righting reflex in mice lasting 13.6 min. At a lower dose (0.1 mg/kg), it reduced spontaneous motor activity in mice by 70.3% and exhibited a synergistic sedative effect when combined with a subthreshold dose of sodium pentobarbital. In canines, RJ-II (**17**) produced synergistic anesthetic effects with chlorpromazine and scopolamine, yielding an average anesthesia duration of approximately 32 min, comparable to pethidine. Additionally, secorhodomollolides B (**185**) and D (**187**) enhanced sleep induction in mice treated with sodium pentobarbital, increasing the sleep rate by 66.7% and 100%, respectively (Wang et al., 2010).

### 6.6 Anti-tumor activity

Early studies reported that extracts of *R. molle* roots prolonged the survival time of mice bearing Walker tumors. The methanol extract of *R. molle* leaves significantly inhibited HT-29 cell viability and migration, induced DNA fragmentation, arrested the cell cycle at the S phase, and triggered apoptosis, as evidenced by upregulation of p53/p21 mRNA and an increased Bax/Bcl-2 expression ratio (Zong et al., 2021). Secorhodomollolide B (**185**) exhibited selective cytotoxicity against the human hepatocellular carcinoma cell line Bel-7402, with an IC_50_ of 0.97 μM (Wang et al., 2010). Collectively, these findings suggest that *R. molle* exhibits antitumor potential and may serve as a source of lead metabolites for anticancer drug development.

### 6.7 Anti-microbial and insecticidal activities

Mollolide A (**224**) demonstrated antiviral activity against Coxsackie B3 virus, with an IC_50_ of 27.7 μM (Li et al., 2013), and activated transcription from the XBP1 upstream promoter in IEC-6, 293T, and RAW264.7 cells (Li et al., 2014). Rhodomollin B (**228**) showed moderate activity against influenza virus A/95-359, with an IC_50_ of 19.24 μM (Li et al., 2016). In addition, five diterpenoids (**14**, **16**, **119**, **227**, **228**) were found to inhibit the growth of *Spodoptera litura larvae*, reducing pupal weight and adult emergence, while disrupting protein and lipid metabolism (Zhong et al., 2004). Diterpenoids (**14**, **227**, **228**) also exhibited cytotoxicity against *Spodoptera frugiperda* (Zhong et al., 2005; Zhong et al., 2006). RJ-III (**16**) displayed insecticidal activity against *Pieris rapae larvae* and *S. litura* (Zhong et al., 2006). Mechanistic studies in *S. litura* indicated that these diterpenoids disrupt Na^+^/K^+^ ion uptake (Yi et al., 2014), induce fluctuations in intracellular Ca^2+^ and pH, and arrest the cell cycle at G2/M phase, thereby inhibiting proliferation (Cheng et al., 2011).

### 6.8 Hepato-protective and reno-protective activities

In a CCl_4_-induced rat model of liver injury, low-dose (*R. molle* flowers, 0.07875 g/kg) reduced serum ALT and γ-GT levels. Integration of metabolomics and network pharmacology suggested that the hepatoprotective effect is mediated through modulation of fatty acid metabolism, particularly linoleic acid and arachidonic acid pathways, likely *via* flavonoid and triterpenoid metabolites (Jiang et al., 2025). In a renal injury model, *R. molle* root extract (5 mg/kg) and RJ-II (**17**) (0.04 mg/kg) significantly ameliorated proteinuria, podocyte injury, and glomerulosclerosis, while inhibiting interstitial fibrosis in ADRN-induced nephropathy mice. RJ-II (**17**) reduced NF-κB p65 phosphorylation, decreased infiltration of CD4^+^ T cells, CD8^+^ T cells, and CD68^+^ macrophages, and downregulated TGF-β1 and p-Smad3 protein expression. These findings suggest that *R. molle* root extract and RJ-II (**17**) effectively mitigate proteinuria and renal injury in ADRN, likely through anti-inflammatory effects and suppression of the TGF-β1/Smad signaling pathway (Qiu et al., 2019).

### 6.9 Others

Protein tyrosine phosphatase 1B (PTP1B) is a key negative regulator of insulin and leptin signaling, making it a potential therapeutic target for type 2 diabetes and obesity. Studies have shown that metabolites (**225**, **226**, **227**, **188**–**190**) exhibit moderate PTP1B inhibitory activity, with IC_50_ values ranging from 22.99 to 32.24 μM, whereas other metabolites showed IC_50_ > 200 μM (Zhou et al., 2018a; Zhou et al., 2018b; Zhou et al., 2017a). These findings suggest a potential application of *R. molle* in managing metabolic disorders.

## 7 Toxicology

**FIGURE 9:**
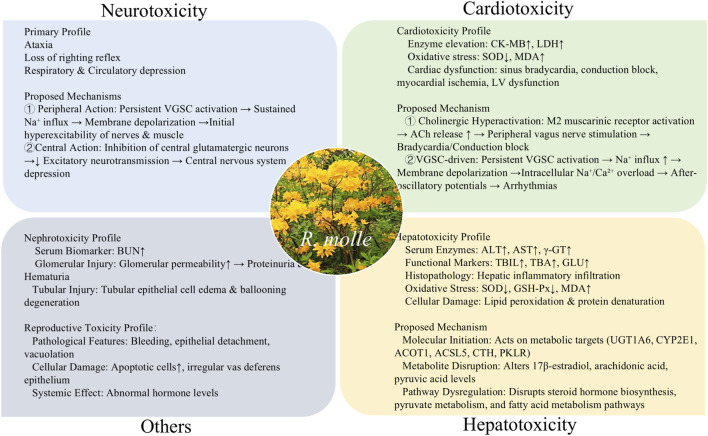
The toxicology of *Rhododendron molle*.

*R. molle* is historically recognized as a highly toxic botanical drug in TCM. Its name, derived from “sheep poisoning,” reflects its neurotoxic effects. Toxicity can manifest rapidly and acutely, including cardiac and neurological symptoms such as hiccups, salivation, hypotension, ptosis, dyspnea, ataxia, hind limb paralysis, and saltatory movements. Severe cases may be fatal due to respiratory failure. Modern toxicological studies confirm its high potency, with a low *LD*_*50*_ of 2.32 g/kg for *R. molle* flowers. Consequently, clinical use is strictly controlled, with a typical dose limited to 0.6–1.5 g (Chinese Pharmacopoeia Commission, 2025). The *LD*_*50*_ of GTX-I (**117**) is only 1.31 mg/kg in mice *via* i.p. administration (Hikino et al., 1976). *R. molle* and certain diterpenoids have been identified as the primary toxic metabolites, with specific LD_50_ values summarized in Table 11. The major toxicological profiles and proposed mechanisms of *R. molle* and its diterpenoid metabolites are illustrated in Figure 9.

**TABLE 11:** The *LD*_*50*_ values of *R. molle* extract and its metabolites.

Phytochemicals	Administration	*LD*_*50*_ values	References
Ethanol extraction of *R. molle* roots	p.o. mice	2,981.457 mg/kg	Zhang (2012)
	p.o. mice	3.491 g/kg	Long (2011)
	p.o. mice	88.54 mg/kg	Yao et al. (2019)
Ethyl acetate fraction of *R. molle* roots	p.o. mice	1908.497 mg/kg	Zhang (2012)
Chloroform fraction of *R. molle* roots	p.o. mice	1,080.341 mg/kg	Zhang (2012)
30% ethanol extraction of *R. molle* roots	p.o. mice	215.62 mg/kg	Yao et al. (2019)
Water extraction of *R. molle* roots	p.o. mice	618.75 mg/kg	Yao et al. (2019)
60% ethanol extraction of *R. molle* flowers	p.o.mice	2.172 g/kg	Guo et al. (2020)
	p.o. mice	1868.58 mg/kg	Wang et al. (2024)
60% ethanol extraction of *R. molle* flowers (vinegar-processed)	p.o. mice	4,650.51 mg/kg	Wang et al. (2024)
	p.o. mice	3.467 g/kg	Guo et al. (2020)
60% ethanol extraction of *R. molle* flowers (wine-processed)
	p.o. mice	4,289.75 mg/kg	Wang et al. (2024)
10% Methanol extract *R. molle* leaves	p.o. rat	96.00 mg/kg	Luo et al. (2023a)
Chloroform fraction of *R. molle* leaves	p.o. rat	279.97 mg/kg	Luo et al. (2023a)
Ethyl acetate fraction of *R. molle* leaves	p.o. rat	239.65 mg/kg	Luo et al. (2023a)
n-Butanol fraction of *R. molle* leaves	p.o. rat	500.09 mg/kg	Luo et al. (2023a)
GTX-I (**117**)	i.p. mice	1.31 mg/kg	Hikino et al. (1976)
	i.p. mice	1.28 mg/kg	Scott et al. (1971)
GTX-III (**10**)	i.p. mice	0.84 mg/kg	Hikino et al. (1976)
	i.p. mice	0.908 mg/kg	Scott et al. (1971)
GTX-II (**107**)	i.p. mice	26.10 mg/kg	Hikino et al. (1976)
RJ-III (**16**)	i.p. mice	0.40 mg/kg	Hikino et al. (1976)
	i.p. mice	271.0 μg/kg	Li et al. (2015)
	p.o. rat	2.35 mg/kg	Yao et al. (2019)
RJ-VI (**118**)	i.p. mice	1.23 mg/kg	Hikino et al. (1976)
	i.p. mice	1791.0 μg/kg	Li et al. (2015)
	p.o. mice	8.47 mg/kg	Yao et al. (2019)
	p.o. rat	10.94 mg/kg	Wang et al. (2024)
RJ-V (**13**)	i.p. mice	0.75 mg/kg	Hikino et al. (1976)
RJ-II (**17**)	i.p. mice	18.50 mg/kg	Hikino et al. (1976)
6-Deoxygrayanotoxin II (**140**)	i.p. mice	>100 mg/kg	Hikino et al. (1976)
RM-LVII (**153**)	i.p. mice	130.90 mg/kg	Song et al. (2025)

### 7.1 Neurotoxicity

In modern pharmacological studies, administration of *R. molle* to mice induced dose-dependent neurotoxicity, characterized by ataxia, sedation, and loss of the righting reflex. Regarding neurotoxicity, some researchers propose that persistent activation of voltage-gated sodium channels (VGSCs) underlies the toxic mechanisms of grayanoids. As early as the 1970s, GTX-I (**117**) and GTX-III (**10**) were shown to reversibly depolarize and activate nerve and muscle membranes in animals, including skeletal muscle in squid axons and guinea pig atria (Seyama, 1970). These toxins increased resting sodium ion permeability, elevated intracellular sodium concentrations due to membrane depolarization (Hotta et al., 1994), and lowered the sodium channel excitation threshold (Narahashi and Seyama, 1974) thereby prematurely activating sodium currents. Studies indicated that nerve axons and muscle fibers across species are sensitive to grayanoid-induced depolarization as long as the membrane generates sodium-dependent action potentials (Starkus and Narahashi, 1978). Persistent VGSC activation is believed to increase vagal tone, contributing to circulatory and respiratory dysfunction (Jansen et al., 2012).

Neurotoxicity is also associated with interactions with the CNS. GTX-I (**117**) significantly enhanced radioactive Na^+^ uptake in meningeal vesicles, suggesting a correlation between toxicity and lipophilicity, which was fully antagonized by TTX (Condrescu and Villegas, 1982). *In vivo*, GTX-I (**117**) and GTX-III (**10**) reversibly inhibited horizontal and vertical motor activity and delayed drug-induced convulsions in a dose-dependent manner, resembling the effects of benzodiazepines (BZPs), including motor inhibition, muscle relaxation, and potentiation of pentobarbital-induced hypnosis (Ohgaki et al., 1987; Ohgaki et al., 1988). Evidence also implicates the γ-aminobutyric acid (GABA) neuronal system in the central inhibitory effects of GTX-III (**10**), as shown by experiments using the GABA agonist barbiturate and antagonist picrotoxin. Additionally, studies using quisqualic acid, a central glutamate receptor agonist, suggest that GTX-III (**10**) may inhibit horizontal movement by modulating central glutamate neurons rather than by activating VGSCs in the brain (Kim et al., 2010; Ohgaki et al., 1988). Furthermore, certain triterpenoids from *R. molle* act as positive allosteric modulators of GABAA receptors (α1β2γ2 subtype), providing another plausible mechanism for CNS-mediated toxicity (Zhang, 2020; Zhang, et al., 2020).

### 7.2 Cardiotoxicity

Clinical cases of *R. molle* poisoning in humans presented with symptoms including shock, arrhythmia, sinus bradycardia, and conduction block. Experimental studies corroborated these findings, showing that extracts of *R. molle* flowers induced myocardial injury in rodents, evidenced by elevated serum CK levels, decreased SOD activity, and increased MDA content, indicating oxidative stress. Specific diterpenoids (**14**, **16**, **17**) caused dose-dependent myocardial injury in rats, manifesting as ST-segment elevation, acute ischemia, left ventricular dysfunction, hemodynamic abnormalities, and elevated LDH and CK-MB levels (Dong et al., 2014).

Similar to neurotoxicity, the cardiotoxicity of grayanoids was primarily attributed to persistent activation of VGSCs in cardiac tissues. GTX-I (**117**) at 0.1–1 μM produced a positive inotropic effect in guinea pig cardiomyocytes, but at a high concentration of 5 μM, it caused arrhythmia or tachycardia. Notably, these positive inotropic and arrhythmic effects were reversible after drug elution or dissociation (Hotta et al., 1994). TTX also antagonized GTX-I (**117**) in a non-competitive manner, increasing resting sodium permeability of squid axon membranes (Narahashi and Seyama, 1974). Previous studies showed that high concentrations of GTX-I (**117**) had no significant effect on Na^+^-K^+^-ATPase. In the Purkinje model of isolated feline heart fibroblasts, grayanoid-induced arrhythmias were mainly triggered by post-oscillatory potentials (Brown et al., 1981). Collectively, these findings suggested that the direct effect of GTX-I (**117**) on myocardial contractility and arrhythmia was mediated by increased resting sodium permeability and generation of persistent action potentials (Ito et al., 1985).

Other studies considered these toxic symptoms similar to cholinergic poisoning, likely related to the cholinergic system. Atropine, a non-specific muscarinic antagonist, ameliorated the bradycardia and respiratory depression induced by grayanoids (Onat et al., 1991). AF-DX 116, a selective M_2_ muscarinic receptor antagonist, eliminated bradycardia—but not respiratory depression—when injected into rats after GTX-I (**117**) administration, suggesting involvement of M_2_ receptors in grayanoid cardiotoxicity (Ohgaki et al., 1988). Moreover, rats given a non-lethal dose of RJ-III (**16**) experienced convulsive death within 30 min when pretreated with huperzine A, an acetylcholinesterase inhibitor. *LD*_*50*_ of RJ-III (**16**) was reduced approximately fourfold after administering 0.5 mg/kg huperzine A 30 min prior, further indicating that cholinergic toxicity was a major limiting factor for these metabolites (Yang et al., 2022b).

### 7.3 Hepatotoxicity

The liver, as the primary organ for xenobiotic metabolism, was highly susceptible to drug-induced injury. As early as 1993, a study reported that administering *R. molle* roots to dogs for 45 consecutive days resulted in elevated serum GPT levels, along with hepatic focal necrosis, hepatocellular edema, ballooning degeneration, and fatty degeneration. Subsequent studies confirmed its hepatotoxicity. Long-term administration of *R. molle* to rats increased serum ALT, AST, and GLU levels, and in mice, it elevated serum ALT accompanied by cytoplasmic vacuolation, hydropic degeneration, hepatic necrosis, inflammatory cell infiltration, and congestion (Guo et al., 2022). Recent studies using a zebrafish larval model demonstrated that 24-h exposure to *R. molle* flowers caused liver tissue damage, apoptosis, and hepatocyte degeneration (Liu et al., 2023). In rats, *R. molle* flowers elicited dose-dependent liver injury, evidenced by increased serum biomarkers (ALT, γ-GT, TBIL, TBA), hepatic oxidative stress (MDA, GSH-Px, SOD), and inflammatory infiltration. Mechanistically, the toxicity was mediated by disruption of steroid hormone biosynthesis, pyruvate metabolism, fatty acid metabolism, and arachidonic acid metabolism *via* targets (UGT1A6, CYP2E1, ACOT1, ACSL5, CTH, PKLR) and their metabolites (e.g., 17β-estradiol, arachidonic acid, pyruvic acid), attributed to diterpenoids such as GTX-III (**10**) and RJ-III (**16**) (Guo et al., 2024; Ran et al., 2025).

Diterpenoids have long been implicated in liver injury. Lipid peroxidation and protein denaturation in mouse hepatocytes were observed at 0.01 mg/kg of GTX-III (**10**) using attenuated total reflection–Fourier transform infrared (ATR-FTIR) spectroscopy (Cakmak-Arslan et al., 2020). Turkish researchers also reported hepatotoxicity in rats following a single large-dose intraperitoneal injection of GTX-I (**117**) (Pischon et al., 2018). One study showed that GTX-I (**117**) and GTX-III (**10**) administered to mice and rats at 1.0 mg/kg and 0.25 mg/kg, respectively, caused weak subchronic toxicity, with pathological examination revealing mild lymphocyte infiltration or microgranulomas in the livers of both rodent species (Hikino et al., 1979).

In contrast to its hepatotoxicity, hepatoprotective effects of *R. molle* have also been documented. In a rat model of CCl_4_-induced liver injury, low-dose *R. molle* flowers (0.07875 g/kg) reduced ALT and γ-GT levels. Integrating metabolomics with network pharmacology suggested that *R. molle* flowers ameliorated liver injury by activating specific pathways and modulating fatty acid metabolism, particularly linoleic acid and arachidonic acid metabolism, likely mediated by flavonoid and triterpenoid metabolites (Jiang et al., 2025). This dichotomy—liver injury *versus* protection—reflected differential outcomes based on dosage, specific bioactive metabolites, and the host’s underlying condition rather than a contradiction.

### 7.4 Nephrotoxicity

Administration of *R. molle* roots to dogs for 45 consecutive days resulted in elevated serum BUN levels, accompanied by increased glomerular permeability, tubular epithelial cell edema, and ballooning degeneration. Complementing this finding, a single high intraperitoneal dose (1 mg/kg) of GTX-I (**117**) induced nephrotoxicity in rats, characterized by proteinuria and hematuria (Pischon et al., 2018).

### 7.5 Reproductive toxicity

After administration of 0.8 μg/kg (i.p.) of GTX-III (**10**), either as a single dose or continuously for 3 weeks, multiple pathological features were observed in testicular tissue, including bleeding, epithelial detachment, irregular vas deferens epithelium, vacuolation, increased apoptotic cells, and abnormal hormone levels (Doğanyiğit et al., 2019).

## 8 Pharmacokinetics

The metabolites of bioactive and toxic diterpenoids from *R. molle* have been investigated over the past decade. Pioneering work established a rapid LC-MS method to investigate the pharmacokinetics of RJ-I (**14**), RJ-II (**17**), RJ-III (**16**) in rats administered *R. molle* flower extracts at different doses. At 21.44 mg/kg, the AUC_0_–t values for RJ-I (**14**), RJ-II (**17**), RJ-III (**16**) was 4.35, 6.09, and 3.15 mg·min/L, respectively, which increased substantially to 56.67, 47.03, and 46.04 mg·min/L at 112.56 mg/kg. Concurrently, the elimination half-lives (T_1_/_2_) were prolonged from 63.65, 133.74, and 83.69 min to 147.57, 215.96, and 219.63 min, respectively (Zou et al., 2014). Subsequently, Zhang et al. developed and validated a specific LC-MS/MS method for RJ-III (**16**) with an improved linear range of 1–200 ng/mL, meeting international standards for precision, accuracy, and stability. Application of this method in mice revealed rapid oral absorption (T_max_ = 0.08 h) and high oral bioavailability (73.6%). RJ-III (**16**) was rapidly eliminated, with T_1_/_2_ values of 0.19 h (intravenous) and 0.76 h (oral), and distributed extensively to the kidney, lung, heart, spleen, and thymus, but minimally penetrated the liver and brain (Zhang et al., 2019).

More recent advances include a UPLC-MS/MS method developed for simultaneous quantification of RJ-II (**17**) and RJ-III (**16**) in rat plasma. The method demonstrated excellent linearity (2–1,250 ng/mL), precision <15%, accuracy of 88%–115%, minimal matrix effects (90%–110%), and recoveries of 78%–87%, confirming its suitability for pharmacokinetic studies (Sun et al., 2024). In a notable study on interaction, an LC-MS/MS method was used to investigate the pharmacokinetics of RJ-II (**17**) and RJ-III (**16**) following administration of *R. molle* flowers alone or in combination with *G. jasminoides*. The method showed good linearity (1–200 ng/mL for RJ-II (**17**), 1–100 ng/mL for RJ-III (**16**) with precision <12% RSD. Co-administration with *Gardenia jasminoides* significantly increased the AUC_0_–t of RJ-II (**17**) (260.44 vs 213.39 h·ng/mL) and RJ-III (**16**) (60.97 vs 22.38 h·ng/mL), prolonged T_1_/_2_ and MRT_0_– ∞ of RJ-II (**17**), and increased the AUC_0_–t, T_1_/_2_, T_max_, and CL of RJ-III (**16**), suggesting that *G. jasminoides* modulated the absorption and elimination of these diterpenoids (Zhang et al., 2023). The above results indicate that the diterpenoids are characterized by rapid absorption and a short half-life. In the context of combined administration, the absorption process was slowed, and the half-life was prolonged. These findings reflect the complexity of the metabolites in TCM and there are complex pharmacokinetic interactions among the metabolites.

## 9 Quality control

*R. molle* is a medicinal plant with significant therapeutic value but considerable toxicity. The current Chinese Pharmacopoeia primarily relied on traditional methods, such as morphology, microscopy, and thin-layer chromatography, for quality control of *R. molle* flowers (Chinese Pharmacopoeia Commission, 2025). To enhance identification accuracy, researchers used oil immersion microscopy to study pollen grain characteristics in detail, providing finer morphological criteria to distinguish it from easily confused species (Chu et al., 2009). However, these methods may not be sufficient to assess the quality of *R. molle* flowers, highlighting an urgent need to incorporate modern analytical techniques to establish more scientific quality standards.

Flavonoids are one of the main metabolite groups. UV spectroscopy was commonly used for rapid determination of total flavonoid content to optimize extraction processes and compare differences across geographical origins and flowering stages (Feng, 2021). More precise HPLC-DAD methods were developed to quantify specific flavonoids, including quercitrin (**295**) and hyperoside (**297**), and revealed dynamic changes in these metabolites across different origins and flowering stages (Feng, 2021). Diterpenoids are the core metabolites responsible for the efficacy and toxicity of *R. molle*. Since diterpenoids lack UV chromophores, universal detectors such as the evaporative light scattering detector (ELSD) or charged aerosol detector (CAD) were often required. HPLC-ELSD methods were established for simultaneous determination of RJ-II (**17**), RJ-III (**16**), and RJ-VI (**118**) (Chen et al., 2020). HPLC-ELSD based fingerprinting combined with pattern recognition analysis was used to compare chemical profiles of roots, flowers, and fruits, confirming the presence of RJ-III (**16**) and RJ-VI (**118**) in all parts, with the highest content in fruits, followed by flowers, and lowest in roots (Song et al., 2022). Furthermore, valued for its superior sensitivity and stability, HPLC-CAD was used for simultaneous quantification of RJ-II (**17**), RJ-III (**16**), and RJ-V (**13**), comparing the content across different origins, harvesting periods, and processed products, providing crucial data for quality evaluation (Feng, 2011; Guo et al., 2020).

Mass Spectrometry (MS), particularly when coupled with chromatography, has significantly enhanced the characterization of the complex chemical profile of *R. molle*. LC-MS has become a pivotal tool for systematic identification of major non-volatile metabolites. A rapid and sensitive HPLC-ESI/QTOF-MS/MS method, coupled with a preliminary tandem solid-phase extraction (SPE) enrichment step, successfully identified 14 diterpenoids from *R. molle* flowers, including RM-I (**109**), GTX-III (**10**), RJ-VI (**118**), RM-XVI (**121**), RJ-III (**16**), rhodomolin B (**111**), kalmanol (**177**), RM-XIII (**116**), RM-III (**120**), RJ-VII (**5**), RJ-II (**17**), and RJ-I (**14**) (Zou et al., 2014). Furthermore, HPLC-TOF-MS analysis identified 25 metabolites, including 9 diterpenoids such as RJ-VI (**118**), kalmanol (**177**), RM-III (**120**), GTX-I (**117**), RJ-III (**16**), GTX-II (**107**), RM-XVI (**121**), RJ-VII (**5**), and RJ-I (**14**). The analysis also revealed significant alterations in the relative peak areas of characterized metabolites in differently processed products (wine- or vinegar-processed), highlighting the impact of processing on the chemical composition of *R. molle* flowers (Wang et al., 2024). Employing UHPLC-Q-Exactive-MS enabled more comprehensive chemical profiling, leading to simultaneous identification of 45 metabolites from the flowers, including 20 diterpenoids and 18 flavonoids (Guo et al., 2024). For the abundant volatile metabolites, GC-MS identified 35 volatiles from an ethanol extract. In contrast, GC-ion mobility spectrometry (IMS), with advantages of speed, high sensitivity, and minimal sample preparation, detected and identified 47 volatile substances in *R. molle* flowers, including 16 alcohols, 14 aldehydes, 9 esters, and 8 other metabolites (Feng et al., 2021). Despite the increasing identification of metabolites for quality control of *R. molle* using technologies such as ELSD, CAD, and MS, currently only two commercially available reference standards—RJ-III (**16**) and RJ-II (**17**)—exist. Considering the content of these two metabolites in the plant, along with their pharmacological effects and toxicity characteristics, it is feasible to use them as quality control standards for the flowers, roots, fruits, and leaves of *R. molle*.

Genomics, transcriptomics, and metabolomics have opened new avenues for understanding the mechanisms underlying quality formation in *R. molle*. Using Illumina sequencing, researchers constructed transcriptome databases for *R. molle* flowers and roots, annotated numerous genes, and identified key enzyme genes (e.g., TPSs and CYPs) potentially involved in diterpenoid biosynthesis (Zhou and Zhu, 2020). Transcriptome analysis of different flower bud differentiation stages preliminarily revealed a potential regulatory role of the SPL gene family in flower bud development (Zhu et al., 2025). Key genes associated with the carotenoid biosynthesis pathway were identified, and their expression patterns correlated with high β-carotene accumulation in the flowers, explaining the molecular basis of yellow flower coloration (Xiao et al., 2018). Recent comparative genomic studies revealed the genetic basis of species-specific genome expansion in *R. molle.* By integrating metabolomics and time-ordered gene co-expression networks, researchers elucidated the distinct molecular regulatory networks underlying yellow flower pigmentation (dominated by dynamic changes in carotenoids, flavonols, and chlorophyll degradation) compared to anthocyanin-based pigmentation in red-flowered species. B3 and ERF transcription factors were identified as key regulators specific to *R. molle* flower coloration (Nie et al., 2023).

## 10 Conclusions and future perspectives

This review systematically summarized comprehensive research progress on *R. molle*, covering its traditional applications, phytochemistry, pharmacological effects, toxicological profiles, pharmacokinetics, and quality control. As an important botanical drug characterized by “efficacy-toxicity duality,” *R. molle* accumulated extensive experience over two millennia of clinical practice, particularly in surgical anesthesia and RA. Modern scientific investigations unequivocally identified diterpenoids as the primary metabolites responsible for both its biological activities and toxic effects. These metabolites demonstrated significant analgesic, anti-inflammatory, immunomodulatory, antihypertensive, antitumor, and anesthetic properties, while also inducing multi-system toxicity affecting neurological, cardiovascular, hepatic, and renal systems. Notably, these bioactive diterpenoids were systemically available in their parent form and exhibited favorable pharmacokinetic properties characterized by rapid absorption and elimination. Regarding quality control, research methodologies evolved from traditional morphological and microscopic identification to advanced analytical techniques, including HPLC-ELSD/CAD, LC-MS, and GC-MS/IMS, for precise quantification of active or toxic metabolites and comprehensive fingerprinting analysis. Furthermore, integration of omics technologies (genomics and transcriptomics) enabled deeper exploration of the biosynthetic pathways and regulatory mechanisms underlying its metabolites.

Despite significant progress, research and application of *R. molle* still faced numerous challenges. First, further elucidation of the precise molecular targets and signaling pathways of key diterpenoids was needed to clarify dose windows and mechanistic differences between their efficacious and toxic effects, providing a theoretical basis for mechanism-based strategies to reduce toxicity and enhance efficacy. Second, current pharmacopoeial standards remained insufficient for comprehensive quality control. Future efforts should integrate multi-omics technologies with chemical analysis to screen key metabolites directly linked to efficacy and toxicity as Q-markers, establish intelligent quality standards covering the entire process from crude botanical drugs to preparations, and incorporate them into pharmacopoeial regulations. Third, traditional processing (with vinegar or wine) was shown to alter chemical profiles and reduce toxicity, but its scientific basis required in-depth analysis. Meanwhile, pharmacokinetic interactions and underlying mechanisms of compatibility with other botanical drugs, such as *G. jasminoides*, should be systematically studied to guide safe clinical use. Finally, potent diterpenoids served as valuable lead metabolites for structural optimization and modification, holding promise for developing novel analgesics or anti-inflammatory agents with higher potency and lower toxicity. Conservation of wild resources and application of advanced technologies, such as synthetic biology, should also be strengthened to achieve sustainable production of rare active metabolites.
